# Comparison of supervised exercise therapy with or without biopsychosocial approach for chronic nonspecific low back pain: a randomized controlled trial

**DOI:** 10.1186/s12891-022-05908-3

**Published:** 2022-11-08

**Authors:** Antonija Hrkać, Darko Bilić, Edita Černy-Obrdalj, Ivan Baketarić, Livia Puljak

**Affiliations:** 1 Faculty of Health Studies, University of Mostar, Mostar, Bosnia and Herzegovina; 2 Rehabilitation Center “Život”, Mostar, Bosnia and Herzegovina; 3 School of Medicine, University of Mostar, Mostar, Bosnia and Herzegovina; 4 Health Center Mostar, Mostar, Bosnia and Herzegovina; 5 Center for Evidence-Based Medicine and Health Care, Catholic University of Croatia, 10 000 Zagreb, Croatia

**Keywords:** Low back pain, Nonspecific, Chronic, Physical therapy, Graded activity, Supervised exercise, Education

## Abstract

**Background:**

A biopsychosocial rehabilitation is recommended for chronic nonspecific low back pain (CNLBP); however, its effectiveness compared to the traditional supervised exercise therapy of CNLBP treatment is still unclear.

**Methods:**

This was a parallel-group randomized controlled clinical trial. The sample consisted of 180 participants of both sexes, aged ≥18 years, with CNLBP for ≥3 months. Using web randomization and concealed allocation, they were assigned to three groups; graded activity receiving cognitive-behavioral therapy, group-based combined exercise therapy and education (GA; *n* = 59), supervised group-based combined exercise therapy and education (SET; *n* = 63), and a control group receiving usual care (*n* = 58). Interventions were administered for 4 weeks (8 sessions). The primary outcome was pain intensity. Outcome measures were collected baseline, after interventions (4 weeks), and during two follow-up periods (3 and 6 months).

**Results:**

After the intervention, GA had a significant large effect on pain reduction compared to the control group (MD of 22.64 points; 95% CI = 16.10 to 29.19; *p* < 0.0001; Cohen’s d = 1.70), as well as SET compared with the control group (MD of 21.08 points; 95% CI = 14.64 to 27.52; *p* < 0.0001; Cohen’s d = 1.39), without significant difference between two intervention groups. At 3 and 6 months of follow-up, GA had a statistically significantly better effect in reducing pain, disability and fear-avoidance beliefs, and improving spinal extensor endurance, range of extension and quality of life compared to SET and the control group. A statistically significantly better effect of SET compared with the control group was found in reducing pain, disability, fear-avoidance beliefs, and improving the physical component of quality of life. Harms were not reported.

**Conclusion:**

This study suggests that graded activity and group-based supervised exercise therapy have beneficial effects over the control group in the treatment of CNLBP. The graded activity was more beneficial than supervised group-based exercise therapy only during the follow-up.

**Trial registration:**

Clinicaltrials.gov (NCT04023162; registration date: 17/07/2019).

**Supplementary Information:**

The online version contains supplementary material available at 10.1186/s12891-022-05908-3.

## Background

Low back pain (LBP) is a major public health problem worldwide; it has been a leading cause of population disability for decades [1]. The most common type of LBP is nonspecific (90–95%), with no identified cause [2]. Chronic nonspecific low back pain (CNLBP), with symptoms present for ≥3 months, has a major impact on global burden and disability; only 10–15% of CNLBP causes 75% of disability and the economic burden of all back pain [3]. In recent decades there has been a significant increase in CNLBP [4]. CNLBP is a complex condition, with the interaction of physical, psychological, and social factors as well as comorbidities [5]. Biopsychosocial treatment that acknowledges and addresses the physical and psychosocial factors underpinning pain and disability is currently considered the most effective approach to chronic pain, including CNLBP [6]. However, gaps between evidence and practice in LBP management have been identified worldwide, implying that many patients receive sub-optimal care [7, 8].

Exercise therapy (ET) is an evidence-based intervention for CNLBP [8, 9]. The main goals of ET are to restore and increase muscle strength and endurance, flexibility and mobility of joints, improvement of balance, coordination and muscle control, and restoration of postural movements and movement patterns. This should reduce pain and disability, leading to faster recovery and return to usual activities. Recent evidence suggests that ET probably reduces pain compared with usual care, no treatment and placebo, and may reduce and improve disability compared with other treatments in chronic LBP [10]. Likewise, evidence shows that ET can improve quality of life, reducing fear of movement, depression and anxiety in the treatment of CNLBP [11, 12].

Several ET interventions were endorsed in clinical guidelines, including supervised exercise therapy (SET), and graded activity (GA) exercises, among others [8].

SET is a well-known physical therapy intervention. It is carried out under the direction and supervision of a physical therapist. It uses a pain-contingent and practice-centered approach, which focus on physical pathology and addressing pain symptoms and physical impairments [13]. There are various types of exercises with different durations and delivery methods [10]. However, no type of exercise was found superior, and there are ambiguities regarding the best dosage and delivery methods [14]. Recent evidence of low quality indicates that stabilization/motor control exercises, resistance exercises, and aerobic exercises are optimal types of ET in reducing pain and disability, and improving mental health and muscle strength in the treatment of CNLBP [15].

GA is a biopsychosocial intervention that consists of combined exercises with a gradual increase in intensity based on quotas that are based on the actual functional capacity of the patient and the ability to meet the goals set in the patient-therapist collaboration. In GA, the approach is time-contingent, instead of pain-contingent, combined with cognitive-behavioral principles, with the main goal to increase activity tolerance through an exercise program during which negative pain-related behavior is neglected, and positive behavior strengthened [16–18]. This type of exercise has shown promising effectiveness in CNLBP [19–22].

Systematic reviews (SR) from 2010 and 2016 reported no difference in the effectiveness of GA and ET in the short, medium, and long-term follow-up periods. Furthermore, there is also limited evidence that GA is more effective than the control group (e.g., usual care) in reducing pain, disability, catastrophizing, and quality of life. However, those findings were based on limited, low-quality and heterogeneous studies, which could have affected the results [23, 24].

Given this state of evidence, the main goal of this study was to compare the effectiveness of SET and GA in the treatment of CNLBP on pain intensity, disability, the range of spinal movement, the strength of the spinal extensor muscles, the degree of depression and anxiety and the quality of life in the period immediately after the intervention (4 weeks), and in the 3rd and 6th month of follow-up.

## Methods

### Study design

This was a parallel-group prospective randomized controlled clinical trial (RCT). This study was approved by the Ethics Committee of the Health Center of Mostar, Mostar, Bosnia and Herzegovina (No: 4458-37-2004/18). Only participants who signed informed consent were included in the study. The study protocol was registered at Clinicaltrials.gov (NCT04023162; registration date: 17/07/2019). All methods were performed in accordance with the Declaration of Helsinki and the trial was reported based on the Guidelines for Consolidated Standards of Reporting Trials (CONSORT).

The study took place from July 2019 to March 2020.

### Participants

Recruitment of participants was conducted over 5 months in the primary medicine setting, among outpatients. Eligible participants were adults of both sexes, aged ≥18 years, suffering from CNLBP lasting ≥3 months and with a feeling of pain most days of the week; diagnosis of CNLBP was confirmed by a family medicine specialist. Participants had to have minimum pain intensity of 40 on the visual analog scale (VAS) ranging from 0 (no pain) to 100 (worst pain). Additionally, participants had to agree to avoid any physical therapy treatments during the study period, except those prescribed by the lead investigator. Eligible participants were either newly diagnosed with CNLBP or those diagnosed with CNLBP in the participating primary care clinic in the last 6 months.

The exclusion criteria were: other subtypes of LBP, history of spine surgery, the presence of other comorbidities (gynecological, vascular, orthopedic, oncological, mental), and participation in an ET program in the past 6 months.

### Randomization and masking

Sequence generation randomization was conducted using the random number generator on the website Randomizer.org (www.randomizer.org). Generated numbers were exported in Microsoft Excel. Allocation concealment was done by a blinded family medicine specialist who determined the participants’ eligibility and enrolled participants in the trial, without knowledge of which code was linked to which intervention.

Implementation: Random sequence was generated by the lead investigator (AH). Three sets of unique numbers, one set for each group, were generated (set #1, set #2 and set #3). The meaning of numerical codes (1, 2, 3 – one code for each group) was known only to the lead investigator. A numerical sequence with the randomized numbers from 1 to 180, divided into three sets, was sent to the family physician, who conducted allocation concealment. The patient considered eligible was assigned a sequential number, and the family physician followed the randomization sequence to see to which numerical code (1–3, or) the patient was assigned. The family physician informed the lead investigator (AH) that a new patient was assigned to a certain numerical code. Then, the lead investigator contacted participants and scheduled participant enrollment, depending on the group in which participants were assigned.

Participants of intervention groups and physical therapists could not be blinded due to the nature of the interventions of ET. To minimize potential bias, participants and physical therapist in the SET group were not provided with more detailed information about the type of intervention in the GA group (type of exercises, study hypothesis); they only knew that the exercises were applied in the other group as well. Measurements were conducted by the outcome assessor (IB), who was blinded to the nature of the intervention, the study hypothesis, and participants’ allocation per group to prevent observer bias during the collection of performance-based measures (range of motion and the strength of the spinal extensor muscle). Observer bias could not be applied to self-reported measures since the participants were evaluators, and they could not be blinded to the interventions.

### Sample size and power calculation

A priori sample size was calculated based on post-treatment results of pain measured with a 100 mm visual analog scale (VAS) from a previously published study [25]. For the sample size calculation, we used the online program at http://www.stat.ubc.ca/~rollin/stats/ssize/, for comparing means for two independent samples; All sample size calculations were checked with the GPower program (Version 3.1.9.4.), using t-test; Means: the difference between two independent means (two groups). Based on the post-treatment results of this study (4 weeks, 3, and 6 months) [25], as well as other studies with a sufficiently similar methodology to our study [16, 26] and the assumption that the minimum difference in pain intensity between intervention groups in post-treatment measurement will be 10 points on VAS, with a standard deviation of 19, and that both intervention groups will achieve a medium effect size in most outcome measures, the study power of 90% and with a dropout rate of 10%, the required sample size is 60 subjects per group.

### Interventions

**Fig. 1:**
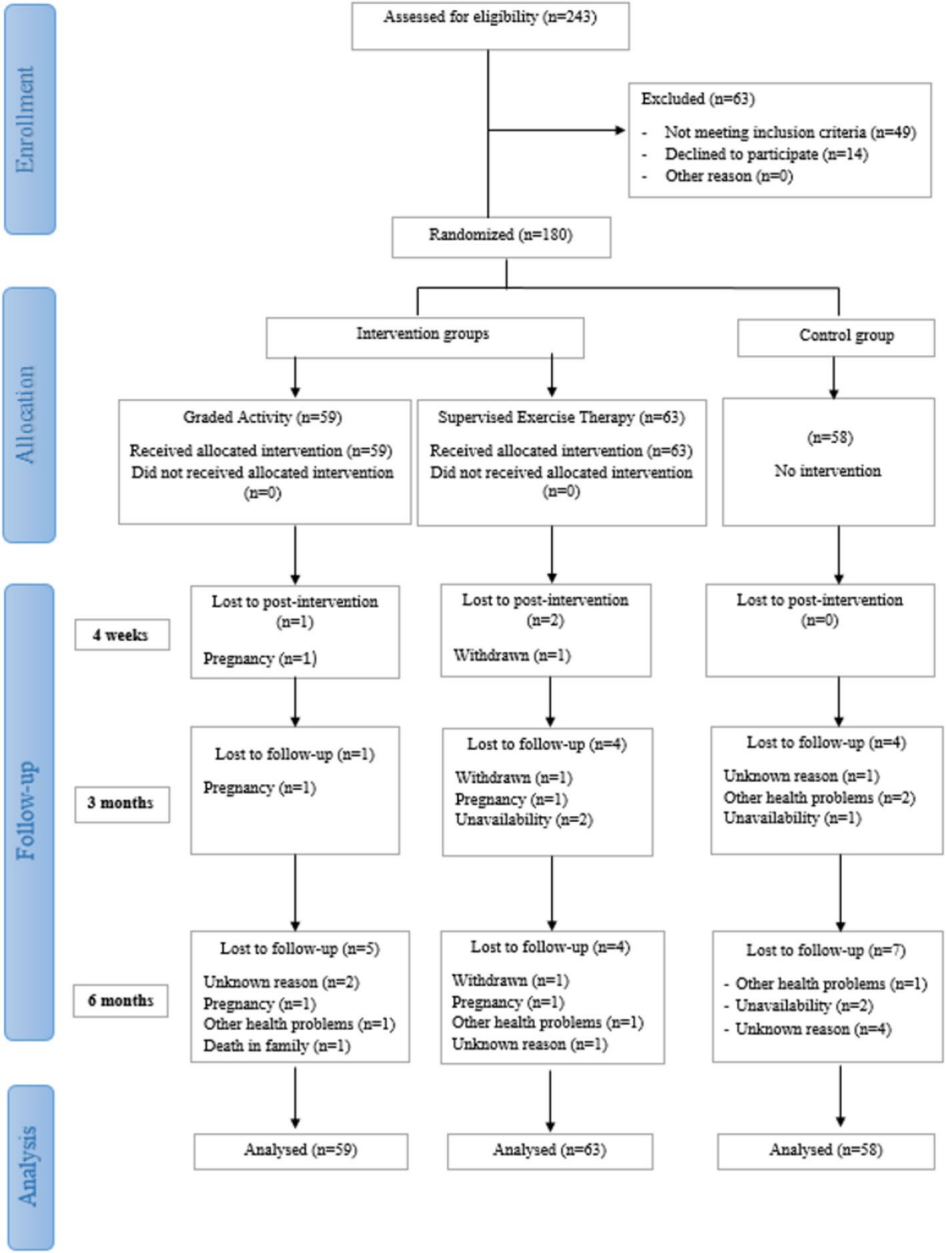
Consort diagram of progress of participants through the study

The total sample consists of 180 participants, divided according to the random number generator into three groups: two intervention groups and one control group (Fig. 1). The first intervention group was the graded activity group (GA; *n* = 59); the intervention consisted of cognitive-behavioral therapy, group-based supervised ET, and education. The second intervention group was a supervised exercise therapy group (SET; *n* = 63), where the intervention consisted of combined supervised group-based ET and education.

The third group was the control group (*n* = 58), which received the usual treatment for CNLBP in the Health Center Mostar, including written and pictorial presentation of recommended TE and optimal posture in some activities. Participants in all groups were asked not to attend other treatments for CNLBP during the study.

Both interventions were carried out at the Rehabilitation Center “Život” in Mostar, Bosnia and Herzegovina. Participants in both intervention groups received 8 sessions over 4 weeks (2 times a week), with one session lasting 60 min, and with the implementation in a group (up to 10 members); according to the recommendations of guidelines from 2009 and in line with the most commonly used in SR from 2017 [12, 27].

All sessions in the intervention groups were delivered by two experienced physical therapists (average experience of 13 years); one for the SET (DB) and another one for the GA (AH). Both physical therapists had postgraduate master’s degree–level qualifications in physical therapy (MPT). The intervention of GA was performed by MPT with current knowledge of back pain and mechanisms of chronic pain; he had positive beliefs about the biopsychosocial approach and the intervention itself. To implement this intervention, he was further educated through the scientific literature and consultation with local psychiatrists [16, 28–30]. Four months before the start of the study, the MPT was practically trained through a pilot study in the implementation of GA among the population with LBP (25 participants).

Likewise, a program of ET was designed according to recommendations and evidence of physical activity and exercise [12, 16, 28, 31]. In addition, a more detailed determination of certain exercises, regardless of which type they belong to, acceptable to the different age populations with CNLBP was also determined through a pilot study among the female population with mixed LBP a few months before the start of the study (30 participants).

In the control group, basic information with written and pictorial instructions for ET and proper posture and movements were delivered by a family medicine specialist (ECO). One physical therapist (IB) participated in the outcome assessment and data collection.

### Graded activity

The intervention in the GA group included three components, cognitive-behavioral therapy, ET implemented under the direction and supervision of an MPT, and education of participants. The intervention was created according to the original description of GA by Lindstrom et al. and a previously published study by Macedo et al., with the deviation in the workplace visits at the request of the participants [16, 28]. The main goal of this intervention is to increase activity tolerance through an exercise program during which behavior in the disease is neglected and positive behavior is strengthened. The program of exercises takes place in a time-contingent manner, starting from the initial assessment of functional capacity and ability to meet the goals set in the patient-therapist collaboration (participants identified problematic activities and movements that they cannot or do not want to perform due to back pain). The activity program consisted of combined types of exercises, same as in the SET group, with deviation in intensity, repetition, and endurance of exercises, individually tailored to each participant depending on functional capacity, functional goals, and “quotas”. The main differences between the two intervention groups are shown in Table 1. A more detailed description of GA is attached in Additional file 1.

**Table 1:** Difference between the interventions received by the two intervention groups

	Graded Activity	Supervised Exercise Therapy
Pain-contingent		✓
Time-contingent	✓	
Ignoring illness behavior and strengthening positive behavior	✓	
Quotas/pacing	✓	
Goal settings	✓	✓
Individually tailored program	✓	
Group tailored program		✓
Group performed (up to 10 members)	✓	✓
Supervised exercises	✓	✓
Pain-free exercises		✓
Types of exercises		
Aerobic (on the Gym ball)	✓	✓
Muscle Stretching	✓	✓
Flexibility and mobility	✓	✓
Muscle strength	✓	✓
Core stabilization	✓	✓
Resistance (with/without dumbbells of 1 kg)	✓	✓
Balance and coordination	✓	✓
Deep breathing	✓	✓
Proper posture pattern	✓	✓
Movement pattern	✓	✓
Education	✓	✓
Home exercises in follow-up	✓	✓

### Supervised exercise therapy group

The intervention in the SET group included two components: supervised group-based ET and education. The program consisted of a combination of ET (aerobics, stretching and flexibility, core, motor control, resistance, balance and coordination, and breathing exercises), with stronger, gradually increased intensity. The intensity of the exercises, the number of repetitions, the duration of the holding phase of the movement during the exercise, and the duration of rest between exercises (or sets) were determined based on the estimated initial intensity and functional capacity (range of motion of anteflexion and extension of the spine and endurance of spinal extensor muscles). The initial intensity of ET was determined according to the heart rate and the Karvonen formula; Instructions for the measurements were taken from a study by Chatzitheodorou et al. [32]. These findings were served for adjustment of exercise intensity to a group (groups formed according to the age of the participants and the similarity of the estimated load and functional capacity). This group is guided by pain; the pain was a limiting factor during the evaluation and implementation of the ET program. Exercises were mostly pain-free. The physical therapist demonstrated each exercise. Goals were set in group-therapist collaboration (reducing pain and increasing mobility).

During the first week, 3 sets of exercises were applied, with 7 repetitions for most exercises, with the hold phase of the exercise of 3 seconds (except for stretching exercises where the hold phase of the exercise was 15 seconds and there were no repetitions), and the rest between exercises (or sets) averaged 15 seconds. Progression was achieved by increasing repetitions (to 10 repetitions), reducing the hold phase (alternating movements up to the maximum range, without holding) and resting between exercises and sets (up to 5 seconds), and using dumbbells (1 kg). The exercises are mostly performed in the supine, prone and four-legged positions, and on a gymnastic ball. A more detailed description of the ET program is attached in Additional file 2. There were no home exercises during the interventions.

### Education

Education is a guidelines-endorsed component along with ET in the treatment of CNLBP [8, 9, 33]. It consisted of basic information on the anatomy of the lumbar spine, potential causes of nonspecific LBP, neurophysiology of pain, resolving misconceptions about LBP/CNLBP, ergonomics, and the importance of staying active [16, 28, 34, 35].

The education was conducted at the beginning of the interventions during the first two sessions lasting about 10 minutes, and at the end of the interventions. At the end of the intervention, a program of home exercises and walking was recommended in both intervention groups.

More detailed descriptions of education are described in Additional file 3.

### Control group

Participants in the control group were not exposed to any physical therapy treatment; they received the usual treatment provided at that institution (pharmacological therapy if needed and advice to stay active). Additionally, the family doctor provided pictorial and descriptive examples of TE and advice on proper posture during the most common activities of daily life (Additional file 4); without more detailed explanations.

### Outcomes and outcome measures

Outcomes were measured at baseline and 4 weeks (post-intervention), 3 months, and 6 months after randomization.

The primary outcome was pain intensity, measured by subjective assessment of pain using the 100 mm VAS, where the beginning of the line indicates “no pain”, and the end of the line is marked with “worst pain”.

#### Secondary outcomes

*Functional disability* was measured by subjective assessment using the Roland Morris Disability Questionnaire (RMDQ), a 24-item questionnaire on activities of daily life. Participants marked the items that they felt had difficulties due to back pain. The questionnaire is scored with the sum of points from 0 to 24, a higher score indicates a higher level of disability [36].

### Range of spinal movement

Spinal extension movement was measured by the *simple goniometer* (ROM). The participant was in an upright starting position with the knees fully extended and the arms behind the neck. The goniometer axis is placed on the crista iliac. The participant was in an upright starting position with the knees fully extended and the arms behind the neck. The goniometer axis is placed on the crista iliac crest, aligned with the midaxillary line. The participant was instructed to slowly and gradually bend directly backwards, as far as possible, without bending the knees.

The participant repeats the extension movement twice, and the maximum range of extension is taken as a measure. All ROM measurements were taken in the afternoon. Measurements are recorded in degrees (°) [37, 38].

Spinal anteflexion movement measured with *Finger-to-Floor Test* (FTF, Thomayer’s Test) - The test was performed so that the participants are in a standing position, with the knees fully extended and the extended hand. The participant received verbal instructions: “Now bend forward with hands extended, while not bending your knees and try to touch the floor with your fingers if you can.”. The participant repeated the bending movement three times (without taking a measurement), and then performed the maximum movement and kept it. The outcome assessor measured the distance of the tip of the middle finger of the right hand from the floor using a centimeter strip. All measurements were taken in the afternoon, at the same time. Measurements are recorded in centimeters (cm) [39].

### Endurance of spinal extensor muscle group

The endurance of the spinal extensor was evaluated with *the Prone Double Straight Leg Raise Test* (PDSRT). The participant is in a pronated (abdominal) position with his legs extended on the floor (mat). The shoulders are vertically positioned relative to the body, with the arms below the forehead. Participants are asked to lift their outstretched legs upwards and maintain this position as far as possible. Time is measured in seconds. Higher results indicate better endurance of spinal extensor muscle [40].

### Quality of life

*The Short form of Health Survey* (SF 12) is a questionnaire to assess the quality of life associated with health status. It consists of 12 questions: two on physical functioning, 2 on the role of physical functioning, one on physical pain, one on general health, one on vitality, 1 on social functioning, 2 on the emotional role, and 2 on mental health. The results are interpreted as physical component score (0–100) and mental component score (0–100). A higher score indicates better health and quality of life [41, 42]. The results were scored manually [43] and verified using an online program (https://orthotoolkit.com/sf-12/).

### Fear of pain and activity

*The Fear-Avoidance Beliefs Questionnaire* (FABQ) measures a patient’s fear of pain and the consequences of avoiding physical activity due to that fear, leading to an increase in negative physical and psychological effects due to back pain. It is divided into two units (work = 5 items and physical activity = 11 items, in both units scores range from 0 to 6), one measuring the association of work with the current feeling of pain in the back pain and the other the correlation of physical activity with the current feeling of pain in the back pain. It consists of 16 items, with a score from 0 to 6. In the physical activity score, 4 items are scored (2–5; total score 0 to 24), and in the work unit, 7 items (6,7,9,10–12, 15; total score 0 to 42). A higher score in both units indicates a greater fear/avoidance presence [44].

### Depression and anxiety

Assessment of the degree of depression and anxiety as measured by the Hospital Anxiety and Depression (HAD) scale. It consists of 14 questions, 7 questions for depression assessment, and 7 for anxiety assessment, and the question and answer period is related to the past week. The answers are scored in four levels from 0 to 3 (0 = not at all, 3 = all the time). The total score can range from 0 to 21 for depression or anxiety. Individuals with a score of 0–7 are not depressed/anxious, 8–10 indicate a borderline condition and 11–21 represent depression/anxiety [45].

At the beginning of the study, participants completed a socio-demographic questionnaire to collect socio-demographic characteristics (all groups). At the end of the study (6-month follow-up), satisfaction with treatment and economic cost-effectiveness were assessed in intervention groups (Description of satisfaction with intervention/economic cost-effectiveness on the Likert scale from 1 = completely dissatisfied to 5 = completely satisfied). On the same questionnaire was the question about the consummation of medicaments during the study, as well as possible visits to other treatments during the study. Both questionnaires were designed for this study (Additional file 5). A detailed description of the outcome measures used can be found in Additional file 6.

Safety information, i.e., any potential adverse events: non-serious (pain, fatigue) and serious adverse events (hospitalization, fractures, cardio-pulmonary overreaction, heart attack, stroke, and death), was also collected from the participants.

### Statistical analysis

An intention-to-treat analysis was used. Data normality was tested through a visual inspection of histograms. Descriptive statistics using mean (standard deviation), median (interquartile rank), or number (percentage) were used to show participant characteristics. Imputation of missing value was performed with the Expectation-Maximization algorithm; a total of 3.60% of data were missing, and the Little test was not statistically significant (*p* = 0.146) [46]. The analysis of data of participants who completed the study and those who were lost during the study by variables of age, sex, baseline pain, and baseline disability was conducted by the Student’s independent t-test. Multivariate repeated analysis was used to investigate the effect of three groups, time (baseline, post-intervention, 3 and 6-month follow-up) and group-time interactions. Univariate ANOVA was used for more detailed information on significant main or interaction effects. Pairwise comparisons with Bonferroni correction were used to compare the baseline to each follow-up. Sensitivity analysis was performed for the primary outcome, intention to treat analysis with or without covariates (sex, age < 40 and ≥ 40, baseline pain, disability score < 14 and ≥ 14, depression score ≤ 10 and ≥ 11) [47]. The results are presented as mean difference (MD), 95% confidence interval (95% CI), and Cohen’s d effect sizes were calculated, where 0.2 was considered a small effect, 0.5 a moderate effect, and 0.8 a large effect [48]. Participants’ satisfaction with the interventions and their economic cost, as well as the use of medications (baseline and after treatment), were tested by the Chi-square test. All statistical analyses were checked by a second statistician. In all tests (two-sided), the significance level was *p* < 0.05. No interim analyses were undertaken during the study period. Statistical software IBM SPSS Statistics 23 (Armonk, NY: IBM Corp.) was used for data analysis.

## Results

Baseline participants’ characteristics are shown in Table 2. There were no significant differences in baseline characteristics between the groups. Participants were mainly female (63.3%), had a mean age of 49.3 (SD 11.7) years, and had experienced CNLBP for a mean of 12.6 (SD 8.1) months.

**Table 2:** Baseline characteristics of participants

Characteristic	Finding in the group N (%)		
	Graded Activity (*n* = 59)	Supervised exercise therapy (*n* = 63)	Control (*n* = 58)
Sex			
Men	25 (42.4)	23 (36.5)	18 (31.0)
Women	34 (57.6)	40 (63.5)	40 (69.0)
Age (*years*)^a^	49.2 (11.6)	48.6 (12.3)	50.2 (11.2)
Height (*cm*) ^a^	175.9 (8.7)	174.1 (8.5)	173.4 (8.9)
Weight (*kg*) ^a^	82.0 (14.6)	80.0 (13.7)	81.5 (14.0)
BMI (*kg/m*^*2*^) ^a^	26.4 (4.1)	26.3 (3.7)	27.0 (3.7)
Marital status			
Single	8 (13.6)	10 (15.9)	7 (12.1)
Married	47 (79.7)	48 (76.2)	43 (74.1)
Divorced	- (−)	- (−)	1 (1.7)
Widow/er	4 (6.8)	5 (7.9)	7 (12.1)
Education level			
Elementary school	- (−)	- (−)	2 (3.4)
High School	43 (72.9)	43 (68.3)	39 (67.2)
College	16 (27.1)	20 (31.7)	17 (29.3)
Employment status			
Student	- (−)	- (−)	- (−)
Employment	50 (84.7)	49 (77.8)	44 (75.9)
Unemployment	(11.9)	6 (9.5)	3 (5.1)
Retired	2 (3.4)	8 (12.7)	11 (19.0)
Type of work			
Mostly sitting job	24 (48.0)	19 (38.8)	16 (39.0)
Hard work^c^	26 (52.0)	30 (61.2)	25 (61.0)
Smoking (*YES*)	19 (32.2)	24 (38.1)	15 (25.9)
Duration LBP (*months*) ^b^	12 (9–18)	10 (6–14)	10 (6–18)
Use of medications (*YES*)	50 (84.7)	56 (88.9)	52 (89.7)
Frequency of use medication			
Daily	14 (28.0)	20 (35.7)	13 (25.0)
Weeks	22 (44.0)	25 (44.6)	23 (44.2)
Months	14 (28.0)	11 (19.6)	16 (30.8)

^a^Data are expressed as mean (standard deviation)

^b^Data are expressed as median (interquartile range)

^c^Jobs which involves moving, standing, and strenuous physical work

In the post-intervention measurement, 3 participants (1.7%) were lost, in the 3-month follow-up 9 (5%) participants, and in the 6-month follow-up 16 (8.9%) participants. The flow of participants throughout the study was shown in Fig. 1. There was no difference in average means between the subgroup of participants lost during the study and the participants who completed the study by age (MD of − 2.52 points; 95% CI − 10.03 to 4.99; *p* = 0.508), baseline pain (MD of 1.15 points; 95% CI − 7.22 to 9.53; *p* = 0.786), as well baseline disability (MD of 2.18 points; 95% CI − 1.48 to 5.83; *p* = 0.242).

### Outcomes

**Fig. 2:**
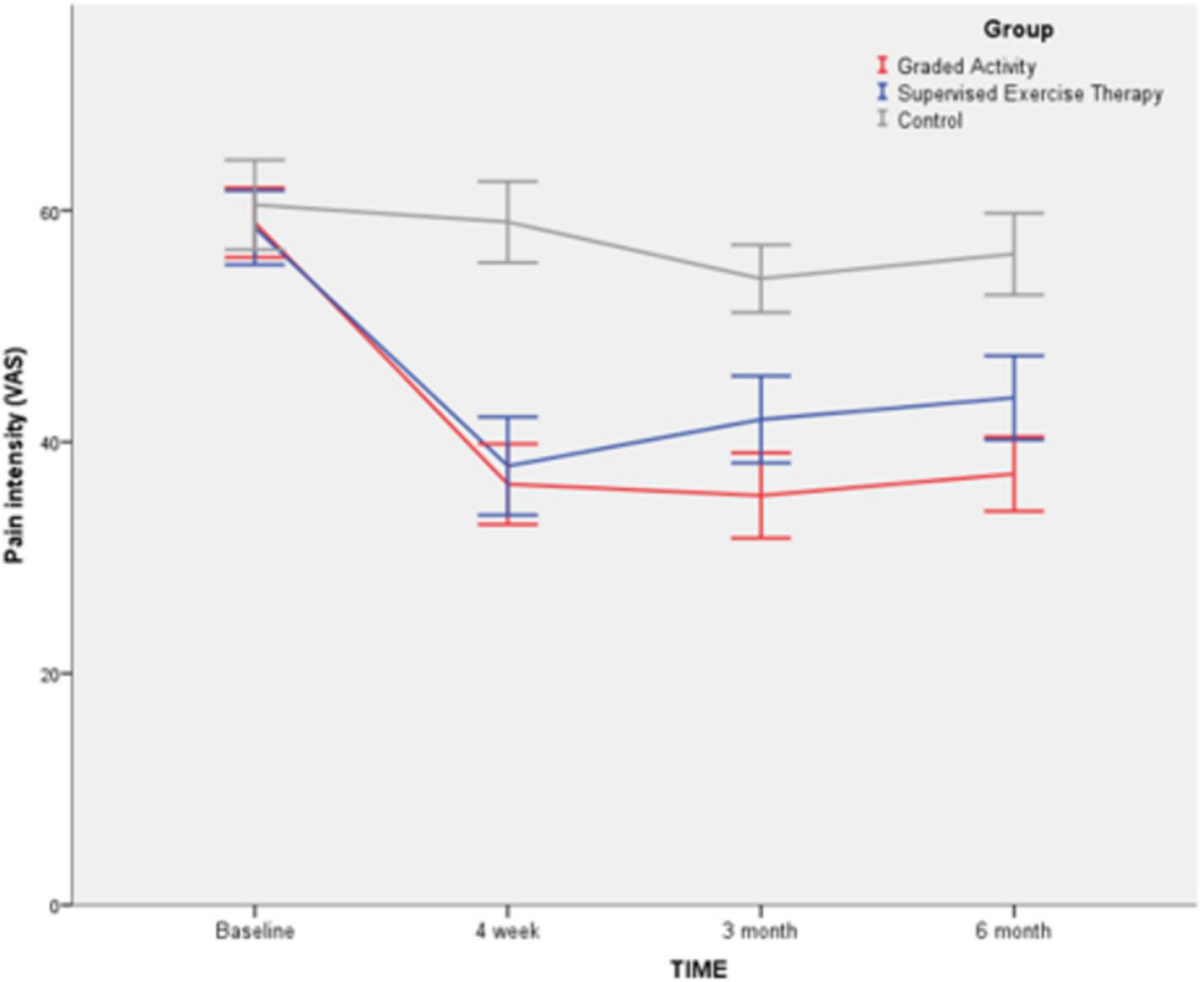
Mean pain intensity (VAS) by group and time. *Error bars show 95% Confidence Interval (CI)

The graph of results of the primary outcome (pain intensity) is shown in Fig. 2. The results of intention-to-treat analysis and sensitivity analyses of the group’s effect on pain intensity at post-intervention, 3 and 6 months follow-up are shown in Table 3. Results of comparisons of secondary outcome measures by groups and time of measures are shown in Table 4.

**Table 3:** Intention to treat analysis—difference between group means (95% CI for difference) for primary outcome

Primary outcome	Graded Activity (*N* = 58)	Supervised Exercise Therapy (*N* = 63)	Control (*N* = 59)	Group comparison					
				GA vs Control		SET vs Control		GA vs SET	
	M (SD)	M (SD)	M (SD)	Mean difference (95% CI)	*p* value‡	Mean difference (95% CI)	*P* value‡	Mean difference (95% CI)	*p* value‡
Pain (VAS; 0 = best, 100 = worst)									
Baseline	58.9 (11.6)	58.5 (12.7)	60.5 (14.7)						
Post-intervention	36.4 (13.4)	37.9 (16.8)	59.0 (13.3)						
Sensitivity analysis^a^				−22.64 (−29.19 to −16.10)	< 0.0001	− 21.08 (−27.52 to − 14.64)	< 0.0001	−1.56 (−7.98 to 4.85)	1.0000
Sensitivity analysis^b^				−19.94 (−25.14 to − 14.74)	< 0.0001	−19.23 (− 24.32 to − 14.13)	< 0.0001	−0.72 (−5.78 to 4.35)	1.000
Effect size (95% CI)^c^				1.70 (1.28 to 2.12)		1.39 (0.99 to 1.79)		0.10 (−0.25 to 0.46)	
3 month	35.4 (14.1)	41.9 (14.9)	54.1 (11.1)						
Sensitivity analysis^a^				−18.73 (−24.78 to −12.68)	< 0.0001	− 12.16 (− 18.11 to −6.21)	< 0.0001	−6.57 (− 12.49 to − 0.64)	0.024
Sensitivity analysis^b^				−16.85 (− 22.25 to − 11.44)	< 0.0001	−10.87 (− 16.17 to − 5.57)	< 0.0001	−5.98 (− 11.24 to − 0.71)	0.020
Effect size (95% CI)^c^				1.47 (1.06 to 1.88)		0.93 (0.55 to 1.30)		0.45 (0.09 to 0.81)	
6 month	37.2 (12.3)	43.8 (14.5)	56.2 (13.4)						
Sensitivity analysis^a^				−19.00 (−25.02 to − 12.99)	< 0.0001	−12.42 (− 18.34 to −6.50)	< 0.0001	− 6.58 (− 12.47 to − 0.69)	0.023
Sensitivity analysis^b^				−17.35 (− 22.89 to −11.81)	< 0.0001	− 11.29 (−16.71 to −5.86)	< 0.0001	− 6.06 (− 11.45 to − 0.68)	0.022
Effect size (95% CI)^c^				1.48 (1.07 to 1.89)		0.89 (0.52 to 1.26)		0.49 (0.13 to 0.85)	

‡ Bonferroni corrected/adjusted *p*-value

^a^Based on intention-to-treat analysis

^b^Based on intention-to-treat analysis and adjustment for age (< 40; ≥40), sex, baseline pain, RMDQ score (< 14;≥14), Depression score (≤10, ≥11);

^c^Cohen’s *d* computed as the mean difference relative to pooled SD of baseline scores; 0.1 was considered a no effect, 0.2 a small effect, 0.5 a moderate effect, 0.8 a large effect

**Table 4:** Intention to treat analysis - difference between groups in secondary outcomes at post-intervention, 3 and 6 months follow-ups

Secondary outcome	Graded Activity (*N* = 58)	Supervised Exercise Therapy (*N* = 63)	Control (*N* = 59)	Group comparison					
				GA vs Control		SET vs Control		GA vs SET	
				Mean difference (95% CI)	*p* value‡	Mean difference (95% CI)	*P* value‡	Mean difference (95% CI)	*p* value‡
RMDQ (0 = best, 24 = worst)									
Baseline	8.7 (4.9)	9.3 (6.0)	10.4 (6.1)						
Post-intervention	4.1 (3.1)	5.0 (4.9)	9.9 (6.2)	−5.81 (−8.02 to −3.61)	< 0.0001***	−4.91 (− 7.08 to − 2.74)	< 0.0001***	−0.90 (− 3.06 to 1.26)	0.939
3 month	3.1 (2.4)	4.7 (4.4)	9.0 (5.7)	− 5.89 (− 7.87 to − 3.92)	< 0.0001***	− 4.33 (− 6.27 to −2.39)	< 0.0001***	−1.57 (− 3.50 to 0.36)	0.154
6 month	3.0 (2.3)	5.5 (4.2)	9.6 (6.0)	−6.59 (− 8.59 to − 4.59)	< 0.0001***	− 4.07 (− 6.04 to − 2.11)	< 0.0001***	−2.52 (− 4.48 to − 0.56)	0.007**
ROM – E (°,higher score best)									
Baseline	19.1 (5.7)	17.8 (5.5)	17.8 (5.8)						
Post-intervention	23.7 (6.5)	20.8 (5.7)	17.7 (5.2)	6.0 (3.3 to 8.6)	< 0.0001***	3.1 (0.6 to 5.6)	0.010**	2.8 (0.3 to 5.4)	0.026*
3 month	24.5 (6.0)	20.6 (5.1)	18.0 (5.4)	6.5 (4.0 to 9.0)	< 0.0001***	2.6 (0.2 to 5.1)	0.031**	3.9 (1.5 to 6.3)	< 0.0001**
6 month	23.8 (6.7)	20.5 (6.1)	17.8 (5.2)	5.9 83.2 to 8.6)	< 0.0001***	2.6 (0 to 5.3)	0.054	3.3 (0.6 to 5.9)	0.009**
FTF (cm, higher score worst)									
Baseline	13.0 (12.0)	12.2 (12.9)	13.1 (13.1)						
Post-intervention	8.2 (9.6)	9.8 (11.9)	13.5 (12.8)	−5.34 (− 10.49 to − 0.19)	0.039**	−3.74 (−8.80 to 1.33)	0.230	− 1.61 (−6.65 to 3.44)	1.000
3 month	6.4 (9.1)	8.9 (11.3)	12.7 (12.5)	−6.27 (− 11.22 to − 1.32)	0.008***	−3.73 (−8.61 to 1.14)	0.197	− 2.53 (−7.39 to 2.32)	0.626
6 month	6.1 (9.3)	9.0 (11.1)	13.2 (12.5)	−7.07 (− 12.00 to − 2.15)	0.002***	−4.18 (−9.02 to 0.67)	0.116	− 2.90 (− 7.72 to 1.92)	0.444
PDSLRT (s, higher score best)									
Baseline	22.5 (25.3)	22.0 (19.8)	21.8 (20.3)						
Post-intervention	98.4 (58.4)	44.8 (32.9)	30.9 (23.3)	67.59 (47.99 to 87.18)	< 0.0001***	13.91 (−26.15 to −1.67)	0.022**	53.67 (33.04 to 74.30)	< 0.0001***
3 month	97.0 (59.3)	40.9 (29.1)	30.4 (23.8)	66.59 (46.69 to 86.48)	< 0.0001***	10.47 (−0.95 to 21.90)	0.080**	56.11 (35.73 to 76.50)	< 0.0001***
6 month	107.1 (73.4)	40.8 (31.3)	23.9 (23.3)	83.18 (59.16 to 107.20)	< 0.0001***	16.84 (5.00 to 28.68)	0.003**	66.34 (41.65 to 91.04)	< 0.0001***
SF-12 Physical (%, 0 = worst, 100 = best)									
Baseline	37.5 (7.4)	38.2 (8.3)	36.2 (8.7)						
Post-intervention	46.7 (6.2)	44.3 (6.8)	36.8 (8.3)	9.97 (6.76 to 13.19)	< 0.0001***	7.53 (4.24 to 10.83)	< 0.0001***	2.44 (−0.36 to 5.25)	0.101
3 month	47.4 (7.1)	44.1 (7.6)	36.5 (7.7)	10.85 (7.60 to 14.10)	< 0.0001***	7.59 (4.29 to 10.89)	< 0.0001***	3.26 (0.10 to 6.42)	0.042**
6 month	46.9 (7.1)	40.6 (7.2)	38.0 (8.5)	8.92 (5.49 to 12.34)	< 0.0001***	2.67 (−0.73 to 6.07)	0.154	6.25 (3.19 to 9.31)	< 0.0001***
SF-12 Mental (%, 0 = worst, 100 = best)									
Baseline	46.6 (9.4)	46.7 (9.4)	45.6 (10.5)						
Post-intervention	52.8 (5.9)	49.8 (8.5)	45.2 (9.1)	7.55 (4.17 to 10.93)	< 0.0001***	4.56 (0.76 to 8.37)	0.014**	2.99 (−0.15 to 6.12)	0.065
3 month	53.6 (6.2)	49.9 (7.8)	46.4 (9.1)	7.19 (3.77 to 10.61)	< 0.0001***	3.51 (−0.15 to 7.18)	0.064	3.68 (0.65 to 6.70)	0.013**
6 month	53.0 (6.2)	46.4 (8.5)	46.2 (8.3)	6.72 (3.48 to 9.95)	< 0.0001***	0.15 (−3.48 to 3.79)	0.994	6.56 (3.37 to 9.76)	< 0.0001***
FABQ Work (0 = best, 42 = worst)									
Baseline	22.3 (5.3)	21.6 (7.3)	21.6 (9.5)						
Post-intervention	14.6 (6.5)	17.5 (8.8)	20.6 (9.7)	−6.06 (−9.71 to −2.41)	< 0.0001**	−3.13 (−7.14 to 0.88)	0.157	−2.93 (−6.23 to 0.37)	0.093
3 month	11.4 (7.9)	18.3 (9.5)	19.8 (9.6)	−8.40 (−12.29 to −4.52)	< 0.0001***	−1.48 (−5.63 to 2.66)	0.673	− 6.92 (−10.68 to −3.15)	< 0.0001**
6 month	11.7 (8.9)	19.3 (9.3)	21.0 (9.2)	− 9.27 (−13.24 to − 5.29)	< 0.0001***	− 1.66 (− 5.66 to 2.34)	0.588	− 7.61 (− 11.52 to − 3.70)	< 0.0001***
FABQ activity (0 = best, 24 = worst)									
Baseline	18.7 (4.4)	18.7 (4.4)	18.6 (5.2)						
Post-intervention	8.5 (5.4)	12.2 (6.6)	18.0 (4.9)	−9.44 (−11.71 to −7.17)	< 0.0001***	−5.81 (−8.30 to −3.31)	< 0.0001**	− 3.63 (−6.21 to − 1.05)	0.003**
3 month	6.5 (6.2)	13.0 (5.6)	17.8 (5.2)	−11.31 (− 13.81 to − 8.80)	< 0.0001***	−4.84 (− 7.15 to − 2.53)	< 0.0001**	−6.47 (− 9.00 to − 3.93)	< 0.0001***
6 month	7.3 (6.2)	14.6 (5.6)	18.2 (4.7)	−10.96 (− 13.37 to − 8.54)	< 0.0001***	−3.62 (− 5.85 to − 1.38)	0.001*	− 7.34 (− 9.88 to − 4.80)	< 0.0001***
HAD Depression (0 = best, 21 = worst)									
Baseline	5.8 (2.4)	5.6 (3.6)	6.0 (5.0)						
Post-intervention	4.2 (2.0)	4.9 (3.4)	6.1 (4.2)	−1.96 (−3.41 to −0.51)	0.005**	− 1.24 (− 2.89 to 0.41)	0.177	−0.72 (− 1.92 to 0.48)	0.333
3 month	4.0 (2.0)	4.8 (3.1)	6.1 (4.1)	−2.04 (−3.49 to −0.60)	0.003**	− 1.21 (− 2.80 to 0.37)	0.169	− 0.83 (− 1.94 to 0.28)	0.180
6 month	4.2 (2.3)	4.9 (2.9)	6.1 (4.1)	− 1.89 (− 3.35 to − 0.42)	0.008**	−1.17 (− 2.71 to 0.36)	0.169	−0.71 (− 1.84 to 0.42)	0.294
HAD Anxiety (0 = best, 21 = worst)									
Baseline	6.3 (2.5)	6.2 (3.9)	6.6 (4.3)						
Post-intervention	5.1 (2.1)	5.5 (3.4)	6.7 (3.3)	−1.69 (−2.92 to −0.46)	0.004**	−1.22 (− 2.68 to 0.24)	0.121	− 0.47 (− 1.69 to 0.75)	0.627
3 month	4.7 (2.0)	5.5 (3.1)	6.5 (3.6)	−1.79 (−3.07 to − 0.52)	0.003**	−1.00 (− 2.45 to 0.44)	0.231	− 0.79 (− 1.90 to 0.32)	0.215
6 month	4.5 (2.2)	5.3 (2.9)	6.6 (3.4)	−2.07 (− 3.33 to − 0.80)	0.001**	−1.22 (− 2.59 to 0.15)	0.091	− 0.85 (− 1.95 to 0.25)	0.166

*Acronyms: VAS* (Visual Analog Scale) – pain, *RMDQ* (Roland Morris Disability Questionnaire) – functional disability; ROM-E – range of lumbar extension, *FTF* (Finger To Floor) – the range of anteflexion motion in lumbar spine, *PDSLRT* (Prone Double Straight Leg Raise Test) - endurance of the lumbar extensor muscle, *SF 12* – Quality of life, *FABQ* (Fear Avoidance Beliefs Questionnaire); *HAD* (Hospital Anxiety and Depression Scale);

‡Bonferroni corrected/adjusted *p*-value

Cohen’s *d* computed as the mean difference divided with pooled SD of baseline scores;

*Small effect size - Cohen’s in range 0.20–0.49;

**Moderate effect size – Cohen’s in range 0.50–0.79;

***Large effect size – Cohen’s d ≥ 0.80

At baseline, there were no significant differences between the groups in the primary outcome and the secondary outcomes. None of the participants reported adverse effects of treatment.

### Primary outcome

Both intervention groups had a large significant effect in reducing pain compared to the control group in all repeated measurements (Fig. 2, Table 3). In the post-intervention measurement, there was no significant difference between the GA and SET in the reduction of pain intensity; a difference between the group was found in 3 and 6 months of follow-up, in favor of GA (Fig. 2, Table 3).

### Secondary outcomes

Significant group-time interaction was also found for all secondary outcomes (*p* < 0.0001).

Both intervention groups had beneficial effectiveness on secondary outcomes compared to the control group; Significant effectiveness of GA was confirmed in all outcomes and repeated measurements. However, more significant effectiveness of SET over the control group was found in the reduction of disability, increase in extension of spine and endurance of extensor spine muscle, the physical component of quality of life, and reduction of fear of activity after the intervention and 3 months of follow-up on (See Table 4 for detailed results).

There was no difference between the intervention groups in the reduction of disability after the intervention and at the 3-month follow-up. However, moderately significant effectiveness of GA over SET was found in the 6-month follow-up. Also, GA had a significantly better efficiency in all repeated measurements in increasing the endurance of the spinal extensor muscles and increasing the extension movement, but not the anteflexion of the spine. (Table 4).

Likewise, more significant effectiveness of GA over SET in the post-intervention measurement was found in the reduction of negative beliefs about the activity (FABQ activity), while in other psychosocial outcomes there was no significant difference. However, in the follow-up period, more significant GA effectiveness was found in both quality of life components (SF 12) and beliefs about how physical activity and work affected their LBP (FABQ) (See Table 4 for detailed results).

In satisfaction with the intervention and economic cost-effectiveness, there was no significant difference between GA and SET (Table 5). Besides, a significant decrease in analgesic consumption was found in both the intervention groups; in GA, at the end of the study, 41.8% fewer participants used analgesics (baseline 84.7% vs. end 42.9%), compared to 32.9% in the SET (baseline 88.9% vs. end 56%); without significant difference between the two groups (χ2 (1) = 0.046; *p* = 0.829).

**Table 5:** Satisfaction with implemented interventions and economic cost-effectiveness in the Graded Activity and Supervised Exercise Therapy group

Survey items	No. (%) of responders in intervention groups (*n* = 92, 75.4%)		
	Graded Activity (*n* = 42)	Supervised Exercise Therapy (*n* = 50)	
*Treatment satisfaction* (YES)	42 (100%)	50 (100%)	
Completely satisfied	36 (85.7%)	38 (76%)	χ^2^ (2) 2.981; *p* = 0.225
Quite satisfied	2 (4.8%)	8 (16%)	
Neither satisfied nor dissatisfied	4 (9.5%)	4 (8%)	
*Economic satisfaction* (YES)	42 (100%)	50 (100%)	
Completely satisfied	37 (88.1%)	40 (80%)	χ^2^ (2) 5.230; *p* = 0.073
Quite satisfied	3 (7.1%)	10 (20%)	
Neither satisfied nor dissatisfied	2 (4.8%)	- (−)	

## Discussion

The results of this study indicate that both intervention groups, GA and SET, were more beneficial than the control group in reducing pain and disability, as well as other outcomes in the treatment of CNLBP. Immediately after the intervention, there was no difference between the two intervention groups in reducing pain and disability. However, in the follow-up period, GA was more beneficial than SET.

A recent Cochrane systematic review (Hayden et al., 2021; 249 studies with exercise interventions) reported moderate-certainty evidence that exercise therapy is probably effective in treating chronic low back pain compared to no treatment, usual care, or placebo for pain. However, the authors concluded that the observed treatment effect of exercise on pain and functional limitation was small and not clinically important. Adverse effects of exercise treatment were seldom reported; they mainly included increased low back pain and muscle soreness [10]. In our study, we found a large effect size of both intervention groups on pain and disability, and participants did not report any adverse effects.

A significant effectiveness of both interventions, GA and traditional exercise therapy, on reducing pain and disability, but with the better clinical effect of GA immediately after interventions (duration 12 weeks, 3 times a week) was reported by Khan et al. in 2014 [49]. However, that study did not measure other outcomes, so other functional and psychosocial outcomes cannot be compared.

Magalhaes et al. reported that there was no significant difference between GA and group-based ET in pain, disability, quality of life and other relevant outcomes immediately after interventions (6 weeks, 2 times a week), as well as at 3 months and 6 months follow-up [26, 50]. However, Magalhaes et al. used fewer exercises in the GA group than in the group-based ET, and the exercises applied to those two groups were different [26, 50].

Several SRs reported that different ET might have a different effect on treatment outcomes in CNLBP [15, 51–53]. It needs to be emphasized that in the original description of the GA method, the choice of activity or ET was made by a physical therapist based on individual patient characteristics [28]. Therefore, potential differences in recruited participants or adherence could explain the variation in reported effectiveness.

The beliefs and attitudes of the therapists who performed the interventions may have a potential impact on the outcome. In recent years, there has been a shift in the field of physical therapy and rehabilitation from a biomedical and practitioner-oriented approach to a biopsychosocial and person-centered approach [29, 54]. In many countries, the biopsychosocial model has become the core model of the physical therapy curriculum. However, it has been reported that physical therapists in some settings have not yet widely adopted a biopsychosocial approach or interventions that include a component of cognitive-behavioral therapy [55]. Physical therapists’ (or healthcare professionals’) beliefs about treatment orientation and fear-avoidance have an impact on clinical practice and advice given to the patient [55].

In our study, GA was performed by experienced MPT with the current knowledge of back pain and pain pathway. Positive beliefs of the biopsychosocial model were assessed with the Pain and Attitudes and Beliefs Scale for Physiotherapists (PABS-PT). The MPT also underwent practical training to implement GA 4 months before the start of the study. We ensured that one MPT performed interventions each, one for SET and the other for GA. Also, to more clearly determine the effectiveness of both intervention groups, we formed a third, “control” group.

We were motivated to conduct this study due to unclear or insufficient evidence about the studied interventions reported in earlier SRs [23, 56]. For example, SR of Van der Gissen et al. from 2012 (10 studies, 680 participants with CNLBP) reported that there is no or insufficient evidence that GA results in better outcomes than usual care [56]. Likewise, SR from 2016 (Lopez-de-Uralde-Villanueva et al.; 6 studies; 1098 participants with CNLBP) reported that in half of the included studies, no difference was found between GA and the control (usual care) group on pain, disability, quality of life and catastrophizing, while in the second half of the included studies more significant effectiveness of GA was found [23].

Education about pain and its assessment, according to the principles of the biopsychosocial approach, improve the therapeutic alliance (therapist-patient relationship), which can also have positive effects on the outcomes in painful conditions [57, 58]. In our study, education about pain was part of both intervention groups, but we do not know how much it affected participants’ perceptions of their CNLBP, as well as the extent to which they continued to exercise regularly in follow-up. The discrepancy between the two groups in the continuation of regular exercise could affect the outcomes.

In any case, comparing GA and traditional exercise therapy requires additional, high-quality studies. During the design of future studies, the attitudes and beliefs of physical therapists should be considered and measured (e.g. The Pain and Beliefs Scale for Physiotherapists). Likewise, as a direction for future studies, Foster and Dellito (2011) state that regardless of advances in understanding the psychosocial aspects of LBP management, several study directions can make a significant contribution in this area. The recommendations include, for example, a clear set of variables to accurately identify patients in need of more intense and comprehensive management, clearly establishing which psychosocial factors are most appropriate as prognostic factors, treatment effect modifiers, and treatment mediators, accurate and systematic psychosocial screening protocols that are feasible for use in clinical practice, evidence on approaches to better target treatment interventions using more defined dosages and high-quality studies that test education strategies at both entry and postgraduation levels [26]. The development and application of clinical prediction rules in physical therapy, and identifying a subset of patients with LBP who are better suited to treatment, can help to improve outcomes [46]. Thus, they could figure out which type of ET is more appropriate for a particular patient with CNLBP.

### Strengths and limitations

This randomized trial had high internal and external validity; in designing our study, we used methods that reduced the risk of bias, and we reported interventions in a detailed and transparent manner. Thus, our interventions should be reproducible. Our results could change the findings of future SR regarding the benefits of biopsychosocial approach in physical therapy. This study also had a high participant completion rate, and it included both short-term and intermediate-term follow-ups. The limitation of the study is the duration of the intervention (4 weeks). Future studies should explore the effect of long-term interventions described in this study. Also, an effect beyond 6 months was not measured in this study. We presented results from the subgroups in results, but we would like to emphasize that the study was not powered for detecting differences between any subgroups. Furthermore, we did not use any outcomes regarding daily activities in our trial; thus, we cannot comment on functional connection with daily activities.

## Conclusion

This study suggests that graded activity and group-based supervised exercise therapy have beneficial effectiveness in the treatment of chronic nonspecific low back pain. However, graded activity has significant effectiveness in the follow-up period. Both interventions were more effective than usual care in patients suffering from chronic nonspecific low back pain. Future trials should investigate which of these two exercises therapy is superior.

## Supplementary Information

**Additional file 1.** Description of Graded Activity.

**Additional file 2.** Program of exercise therapy in Supervised Exercise Therapy group and Graded Activity group.

**Additional file 3.** Education intervention in Graded Activity and Supervised Exercise Therapy groups.

**Additional file 4.** Recommendations of home-performance therapy exercises and instructions on proper posture during daily life activities in the control group.

**Additional file 5.** Sociodemographic Questionnaire and Treatment Satisfaction Questionnaire.

**Additional file 6.** Outcome measurement instruments.

## Data Availability

The datasets used and/or analyzed during the current study are available from the corresponding author on reasonable request.

## Abbreviations

LBP: Low Back Pain
CNLBP: Chronic Nonspecific Low Back Pain
MPT: Master of Physical Therapy
ET: Exercise Therapy
GA: Graded Activity
CBT: Cognitive Behavioral Therapy
SR: Systematic Review
RCT: Randomized Controlled Trial
VAS: Visual Analog Scale
RMDQ: Roland-Morris disability questionnaire
FTF: Finger to the Floor test
ROM-E: Range of extension spinal movement
PDSLRT: Prone Double Straight Leg Raise test
HAD: Hospital Anxiety and Depression scale
SF 12: Health Survey – short form
ANOVA: Analysis of Variance
M: Mean
SD: Standard Deviation
MD: Mean difference
CI: Confidence interval
