# Supplementary material for: Comparison of supervised exercise therapy with or without biopsychosocial approach for chronic nonspecific low back pain: a randomized controlled trial
Source: BMC Musculoskelet Disord. 2022 Nov 8;23:966. doi: 10.1186/s12891-022-05908-3 (PMC9641911; doi:10.1186/s12891-022-05908-3)
Supplement: Supplementary file 1 — Additional file 1. Description of Graded Activity. [file 12891_2022_5908_MOESM1_ESM.docx]

Additional file 1. *Description of Graded Activity*

Adaptation of biopsychosocial intervention was performed according to the original description of graded activity by Lindstrom et al (1992) and previously published by Macedo et al (2012).

According to the original description (Lindstrom et al, 1992), the graded activity consists of 4 components: 1) functional capacity assessment; 2) an individual, submaximal, gradually increased exercise program, with a cognitive-behavioral approach, based on the results of the tests and the demands from the patient's work; 3) a work-place visit; and (3) back school education. The main goal of this intervention is to increase activity tolerance through an exercise program during which behavior in the disease is neglected and positive behavior is strengthened. The program of exercises takes place in a time-contingent manner, starting from the initial assessment of functional capacity and ability to meet the goals set in the patient-therapist collaboration.

An exercise program should be adapted to the patient, his functional capacity, and observed physical work demands; the program may include different types of exercises and activities, exercises with a known benefit in low back pain, and with simple equipment. Following these recommendations, the program of exercise therapy in graded activity is adapted to the current evidence in science on the optimal type/s of therapy exercises for CNLBP, as well as the available conditions in our geographical area. Also, the assessment of functional capacity is adjusted to the available conditions in our geographical area (simple equipment).

Functional goals and “quotas” of the exercise were established in individual contact with each participant (one session, 60 minutes). Assessment of functional capacity included: i) range of lumbar spine anteflexion (FTF test), range of motion of spinal extension (goniometer), and endurance of spinal muscle extensors (PDSLRT). Based on this assessment and established functional goals (performing activities according to the participant’s wishes and fear of activity) and certain “quotas” (exercise intensity, repetition, and endurance for each exercise), participants were assigned to a group with the same and/or similar characteristics. The initial “quota” was set lower than estimated (about 75%) to encourage positive reinforcement during the intervention. During the first weeks of intervention, implementation of exercises was set at 50% of the participants ’initial quote assessment, during the second week at 60%, and during the third and fourth weeks, it was increased to 75%. Quotas were systematically increased; the increase in quota depends on the participant or subgroup, the assessment of the physical therapist, and the participants ’approval (pacing up). A reward-positive reinforcement followed each increase by a physical therapist (e.g., verbal praise). The break between exercises depended on the quota (e.g. the break depended on the performance of a given exercise quota; the full quota had to be achieved for each exercise). Participants were instructed to perform an agreed amount, no less or more, regardless if they felt they were capable of doing more.

At the beginning of the exercise program, the physical therapist demonstrated each exercise. During the exercise session, the physical therapist monitored each participant and recorded participants' complaints of pain and disability, as well as behavior in pain; without changing the program and without paying much attention to complaints. In the event of avoiding the implementation of an exercise or failing to meet a certain “quota,” the physical therapist did not implement the reward, nor did it change the exercise; on the contrary, through verbal communication and assisted movement, the physical therapist encouraged the participant to perform the activity. However, the presence and attention of the physical therapist to the participant/s were gradually reduced during the intervention; which also has the effect of increasing quotas and independence of participants in the performance of exercises.

In this group, during the intervention, we did not visit participants ’workplaces (at the request of participants); ambiguities and job-related inquiries were addressed through education during the intervention.

The activity program consisted of combined therapy exercises, the same as in the physiotherapy group, with deviation in the number of repetitions and intensity of exercises, depending on an assessment of individual functional capacity, functional goals, and quota.

There were no home-exercise programs during the intervention.
