# Supplementary material for: Comparison of supervised exercise therapy with or without biopsychosocial approach for chronic nonspecific low back pain: a randomized controlled trial
Source: BMC Musculoskelet Disord. 2022 Nov 8;23:966. doi: 10.1186/s12891-022-05908-3 (PMC9641911; doi:10.1186/s12891-022-05908-3)
Supplement: Supplementary file 2 — Additional file 2. Program of exercise therapy in Supervised Exercise Therapy group and Graded Activity group. [file 12891_2022_5908_MOESM2_ESM.docx]

*Additional file 2*. Program of exercise therapy in Supervised Exercise Therapy Group and Graded Activity group

The exercise therapy program (ET) was designed after several months of a pilot study in the community (female), on people with mixed symptoms of back pain, emphasizing that in this program exercises were selected primarily for the lumbar spine and according to the scientific literature. The ET program was prepared before the beginning of the implementation of the interventions, with the possibility to modify and adapt the ET to the participant or participants (in a supervised exercise group), as well as principles of interventions.

Note: In both groups, the physical therapist demonstrated each exercise. Each exercise was done in 3 sets.

Before starting the ET, the participants will be taught about proper breathing and how to apply it during exercise therapy.

*Deep Breathing exercise* - Maximum inhalation to the nose (feel the movement of the chest upwards and the abdomen downwards), short holding of the breath, and then exhalation to the mouth (or nose); exhalation lasts longer than inhalation.

Deep breathing exercises are applied at the beginning of the ET session, during rest between exercises, at the end of the ET session and, if possible, during the implementation of another type of exercise (first two weeks).

*Breathing during the movement* - inhaling begins with the movement, holding the breath with the performed movement, and exhaling with the return of the movement to the starting position. The physical therapist used verbal instructions to warn about the application of learned breathing during exercise (mainly during the first two weeks of the ET program).

At the beginning of the intervention, the physical therapist presented the participants with basic information about the ET, its importance, and expected improvement if applied regularly.

*Assessment of initial intensity and functional capacity in the supervised exercise therapy group*

The intensity of the exercises, the number of repetitions, the duration of the holding phase of the movement during the exercise, and the duration of rest between exercises (or sets) in the supervised exercise therapy group were determined based on the estimated initial intensity and functional capacity. The initial intensity of ET was determined according to the heart rate and the Karvonen formula. Before the start of the intervention, through one group session (60 minutes), the participants were shown how to measure the pulse to assess the heart rate (the measurements were carried out over three mornings), a functional capacity was measurement (range of motion of anteflexion and extension of the spine and endurance of spinal extensor muscles); these findings did not serve for individual adjustment of exercise intensity to each participant but a group (groups formed according to the age of the participants and the similarity of the estimated load and functional capacity).

*The exercise therapy program*

**Week 1**

1. Warm-up exercises on a gymnastic ball - 15 minutes (exercises are performed in a sitting position on a gymnastic ball, with a slight bouncing on the ball and alternating movements of the extremities).
2. Supine position, with your knees bent and your arms resting on your body

Exercise 1- arms cover the knee of one leg and gently pull the chest (to the limit of pain), hold for 3 seconds and return to the starting position (*Knee to chest exercise*). Then repeat the same with the other leg. Repeat 7 times with each leg.

Exercise 2 - Lifting the pelvis, holding the contraction for 3 seconds, and returning to the starting position (*Pelvic tilt exercise*). Repeat 7 times.

Exercise 3 - Supine position, knees bent and with support on the feet. Keep your hands in line with your shoulders or above your head. Gently turn both knees symmetrically to the left to the floor, and head to the opposite side of the direction of the knees. Hold the position until the tension loosens (average 15 seconds). Repeat the movement by turning the knee to the right and the head to the left, and keep the movement again (*Trunk rotation exercise*). Repeat 7 times.

Exercise 4 - straightening (back extension) on a gymnastic ball. Supine position on the ball, support on legs bent at the knees. Stretch your legs slightly, until your whole body is stretched (on the ball). Keep your arms close to your body or fully extended above your head. Hold the position for 3 seconds and repeat alternating forward and backward movements up to 7 times.

1. Prone position

Exercise 1- arms placed next to the body, performing partial or complete straightening of the arms (extension). Hold the movement for 3 seconds. Return to starting position in the same way. Repeat 7 times.

Exercise 2 - arms outstretched above the head, lift one arm and the opposite leg together, and alternate (*Alternating Superman exercise*). The movements are performed lightly, holding the contraction for 3 seconds, and repeating 7 times.

Exercise 3 - arms outstretched above the head, raising the arms and legs in an upward direction (*Superman Exercise*), holding the movement (about 3 sec), and repeating 7 times.

Exercise 4 - prone position on a gymnastic ball, supporting hands on the floor, legs outstretched. Pull the body forward on the ball, at the same time creating support on the bent knees on the ball and lifting the pelvis to a flexion movement in the hip and spine (*Hipp raise exercise*). Hold 3 sec and pull back to the starting position. Repeat 7 times.

Exercise 5 – prone position on a gymnastic ball, support hands on the floor, support on the stomach, legs outstretched at the hips and knees (*Leg raise exercise*). Point the extended legs up (as much as the pain allows), hold for 3 seconds, and repeat 7 times.

Exercise 6 - Lean on your elbows, slowly move your head back and forth, and turn to the left and right. Hold each movement for 3 seconds and repeat 7 times.

1. Four-legged position

Exercise 1 - pulling the abdomen up and opposite down (*Cat-Cow exercise*). Hold the movement for 3 seconds, 7 repetitions.

Exercise 2 - alternately raising one arm and the opposite leg, holding the movement for 3 seconds, 7 repetitions (*Quadruped Arm/Leg Raise Exercise*).

Exercise 3 - from the four-legged position, direct the body backward, with outstretched arms, and hold the position for at least 15 seconds (*Prayer exercise*).

**Week 2**

1. Warm-up exercises on a gymnastic ball - 15 minutes (exercises are performed in a sitting position on a gymnastic ball, with a slight bouncing on the ball and alternating movements of the extremities).
2. Exercises on a gymnastic ball

*Exercise 1* - Supine position on the ball, support on legs bent at the knees (*Back extension stretch*). Stretch your legs slightly, until your whole body is stretched (on the ball). Keep your arms close to your body or fully extended above your head. Hold the position for 3 seconds and repeat alternating forward and backward movements up to 10 times.

*Exercise 2* - prone position on a gymnastic ball, supporting hands on the floor, legs outstretched. Pull the body forward on the ball, at the same time creating support on the bent knees on the ball and lifting the pelvis to a flexion movement in the hip and spine (*Decline Plank exercise*). Hold for 3 sec and pull back to the starting position. Repeat 10 times.

Exercise 3 – prone position on a gymnastic ball, support hands on the floor, support on the stomach, legs outstretched at the hips and knees (Hipp raise exercise). Point the extended legs up (as much as the pain allows), hold for 3 seconds, and repeat 10 times.

*Exercise 4* - Standing position, embrace the ball with outstretched arms, and place it at shoulder level. Directing movements above the head. Hold the movement for 3 seconds and repeat 10 times.

*Exercise 5*- standing position, holding the ball in front of the body with outstretched arms and directing it over the head to one side and then the other. Hold the movement for 3 seconds and repeat 10 times.

*Exercise 6* –supine position, legs placed on the ball (bent at the knee). Lifting the pelvis (*Pelvic tilt exercise*). Hold the movement for 3 seconds and repeat 10 times.

1. Supine position, with your knees bent and your arms by your body.

*Exercise 1*- arms cover the knee of one leg and gently pull towards the chest (to the limit of pain), hold for 3 seconds and return to the starting position (*Knee to chest exercise*). Then repeat the same with the other leg. Repeat 10 times with each leg.

*Exercise 2* - lifting the pelvis, holding the movement for 3 seconds, and returning to the starting position (*Pelvic tilt exercise*). Repeat 10 times.

*Exercise 3* - turn the knee left and right and turn the head in the opposite direction from the knee (*Trunk rotation exercise*). Perform the exercises lightly, holding each movement for 3 seconds and repeating them 10 times.

*Exercise 4* - outstretched arms pointing upwards, then touching the knees with the palms (*Partial curl exercise*). Hold for 3 seconds and repeat 10 times.

*Exercise 5* - arms placed behind the neck, legs bent at the knee. Direct the elbow of one hand towards the knee of the opposite leg. Hold each movement for 3 seconds and repeat 10 times.

*Exercise 6* - outstretched legs and outstretched arms above the head. Direct the arms and legs symmetrically towards the stomach. When moving, do not bend your arms at the elbows and legs at the knees. Make sure that the knees do not bend, return the legs fully extended towards the initial position as much as possible, and do not touch the floor. Hold the movement for 3 seconds and repeat 5 times.

1. Prone position

*Exercise 1*- Outstretched arms placed next to the body. Lift your hands slightly from the surface and, fully extended, direct them towards the center of your head (put your palms together). Return to starting position in the same way. Hold the movement for 3 seconds and repeat 10 times.

*Exercise 2* - arms outstretched above the head, lift one arm and the opposite leg together, and alternate (*Alternating Superman exercise*). The movements are performed lightly, holding the contraction for 3 seconds, and repeating it 10 times.

*Exercise 3* - arms outstretched above the head, raise the arms and legs together in an upward direction (*Superman Exercise*). Hold the movement for 3 seconds and repeat 10 times.

*Exercise 4* - prone position on a gymnastic ball with support on outstretched arms and thighs (or knees). Direct the upper part of the body toward the floor (*Push-Ups Exercise*). Repeat 10 times.

Exercise 5 - from the same position (exercise 4), by moving the base of the support on the ball from the abdomen to the thighs, lift the pelvis while moving the ball (bending the back), to the limit of pain (Hipp raise exercise). Repeat 10 times.

Exercise 6 - lean on your elbows, direct your head back and forth, and to the left and right. Hold each movement for 3 seconds and repeat 10 times.

1. Four-legged position

*Exercise 1* - pulling the abdomen up and vice versa down (*Cat-Cow Exercise*). Hold the movement for 3 seconds, 10 repetitions.

*Exercise 2* - alternately raising one arm and the opposite leg, holding the movement for 3 seconds, 10 repetitions.

*Exercise 3* - from a four-legged position (knee support - arms) direct the body backward, with outstretched arms and hold the position for at least 15 seconds (*Prayer Exercise*). From this position, pull the body forward, with greater support on outstretched arms and straightening the back as much as possible. For an additional stretch, point your head back. Hold the movement for 3 seconds and repeat 10 times.

**Week 3**

1. Warm-up exercises on a gymnastic ball - 10 minutes (exercises are performed in a sitting position (correct posture) on a gymnastic ball, with light bouncing on the ball and alternating and simultaneous movement of the extremities.
2. Exercises on/with a gymnastic ball

*Exercise 1 -* Supine position on the ball, support on legs bent at the knees (*Back Extension Exercise*). Stretch your legs slightly, until your whole body is stretched (on the ball). Keep your arms close to your body or fully extended above your head. Perform the movement alternately, without stopping and repeat alternating forward and backward movements up to 10 times.

*Exercise 2 -* prone position on a gymnastic ball, supporting hands on the floor, legs outstretched. Pull the body forward on the ball, at the same time creating support on the bent knees on the ball and lifting the pelvis to a flexion movement in the hip and spine (*Hipp raise exercise*). Perform the movement alternately, without stopping, and repeat alternating forward and backward movements up to 10 times.

*Exercise 3 –* prone position on a gymnastic ball, support hands on the floor, support on the stomach, legs outstretched at the hips and knees. Point the extended legs up (as much as the pain allows) (*Leg Raise Exercise*). Perform the movement alternately, without stopping, and repeat alternating forward and backward movements up to 10 times.

*Exercise 4 -* Standing position, embrace the ball with outstretched arms, and place it at shoulder level. Directing movements above the head. Perform the movement alternately, without stopping and repeat alternating forward and backward movements up to 10 times.

*Exercise 5-* standing position, holding the ball in front of the body with outstretched arms and directing it over the head to one side and then the other. Perform the movement alternately, without stopping and repeat alternating forward and backward movements up to 10 times.

*Exercise 6 –* supine position, legs placed on the ball (bent at the knee). Lifting the pelvis (*Bridge Hill Exercise*). Perform the movement alternately, without stopping, and repeat alternating forward and backward movements up to 10 times.

1. Supine position, with your knees bent and your arms resting on your body

*Exercise 1*- arms cover the knee of one leg and gently pull towards the chest (to the limit of pain), hold for 3 seconds and return to the starting position (*Knee to chest exercise*). Then repeat the same with the other leg. Repeat 10 times with each leg.

*Exercise 3* - Turn the knee left and right and turn the head in the opposite direction from the knee (*Trunk Rotation Exercise*). Perform the exercises lightly, repeating them 10 times.

*Exercise 4* - outstretched arms pointing upwards, then touching the knees with the palms (*Partial curl*). Perform the movement alternately, without stopping and repeat 10 times.

*Exercise 5* - arms placed behind the neck, legs bent at the knee. Direct the elbow of one hand towards the knee of the opposite leg. Perform the movement alternately, without stopping and repeat 10 times.

*Exercise 6* - outstretched legs and outstretched arms above the head. Direct the arms and legs symmetrically towards the stomach. When moving, do not bend your arms at the elbows and legs at the knees. Make sure that the knees do not bend, return the legs fully extended towards the initial position as much as possible, and do not touch the floor. Perform the movement alternately, without stopping. Repeat 5 times.

1. Prone position

*Exercise 1*- Outstretched arms placed next to the body. Lift your hands slightly from the surface and, fully extended, direct them towards the center of your head (put your palms together). Return to starting position in the same way. Perform the movement alternately, without stopping. If possible, perform the exercise with weights (1 kg). Repeat 10 times.

*Exercise 2* - arms outstretched above the head, lift one arm and the opposite leg together, and alternate (*Alternating Superman exercise*). If possible, perform the exercise with weights (1 kg). Perform the movement alternately, without stopping. Repeat 10 times.

*Exercise 3* - arms outstretched above the head, raise the arms and legs together in an upward direction (*Superman Exercise*). If possible, perform the exercise with weights (1 kg). Perform the movement alternately, without stopping. Repeat 10 times

*Exercise 4* - leaning on your elbows, moving your head back and forth, and turning to the left and right. Hold each movement for 3 seconds and repeat 10 times.

1. Four-legged position

*Exercise 1* – alternately raising one arm and the opposite leg, holding the movement for 5 seconds, 10 repetitions. Hold a 1 kg weight in your hands.

*Exercise 2* - pulling the abdomen up and vice versa down (*Cat-Cow Exercise*). Hold the movement for 3 seconds, 10 repetitions.

*Exercise 3* - from the four-legged position, direct the body backward, with outstretched arms and hold the position for at least 15 seconds (*Prayer Exercise*). From this position, pull the body forward, strengthening the armrests and straightening the back as much as possible. For an additional stretch, point your head back. One repeat.

**Week 4**

1. Warm-up exercises on a gymnastic ball - 10 minutes (exercises are performed in a sitting position on a gymnastic ball, with a light bounce on the ball and alternating and simultaneous movement of the extremities).
2. Exercises on/with a gymnastic ball

*Exercise 1 -* Supine position on the ball, support on legs bent at the knees (Back extension. Stretch your legs slightly, until your whole body is stretched (on the ball). Keep your arms close to your body or fully extended above your head. Perform the movement alternately, without stopping and repeat alternating forward and backward movements up to 10 times.

*Exercise 2 -* prone position on a gymnastic ball, supporting hands on the floor, legs outstretched. Pull the body forward on the ball, at the same time creating support on the bent knees on the ball and lifting the pelvis to a flexion movement in the hip and spine. Perform the movement alternately, without stopping and repeat alternating forward and backward movements up to 10 times.

*Exercise 3 –* prone position on a gymnastic ball, support hands on the floor, support on the stomach, legs outstretched at the hips and knees. Point the extended legs up (as much as the pain allows). Perform the movement alternately, without stopping and repeat alternating forward and backward movements up to 10 times.

*Exercise 4 -* Standing position, embrace the ball with outstretched arms, and place it at shoulder level. Directing movements above the head. Perform the movement alternately, without stopping and repeat alternating forward and backward movements up to 10 times.

*Exercise 5-* standing position, holding the ball in front of the body with outstretched arms and directing it over the head to one side and then the other. Perform the movement alternately, without stopping and repeat alternating forward and backward movements up to 10 times.

*Exercise 6 –* supine position, legs placed on the ball (bent at the knee). Lifting the pelvis. Perform the movement alternately, without stopping and repeat alternating forward and backward movements up to 10 times.

1. Supine position, with your knees bent and your arms resting on your body

*Exercise 1*- arms cover the knee of one leg and gently pull towards the chest (to the limit of pain), hold for 3 seconds and return to the starting position. Then repeat the same with the other leg (*Knee To Chest Stretch*). Repeat 10 times with each leg.

*Exercise 2* - lifting the pelvis, holding the contraction for 5 seconds, and returning to the starting position (*Pelvic Tilt*). Repeat 10 times.

*Exercise 3* - Turn the knee left and right and turn the head in the opposite direction from the knee (Trunk rotation). Perform the exercises lightly, repeating them 10 times.

*Exercise 4* - outstretched arms pointing upwards, then touching the knees with the palms (*Partial Curl*). Perform the movement alternately, without stopping and repeat 10 times.

*Exercise 5* - arms placed behind the neck, legs bent at the knee. Direct the elbow of one hand towards the knee of the opposite leg. Perform the movement alternately, without stopping and repeat 10 times.

*Exercise 6* - outstretched legs and outstretched arms above the head. Direct the arms and legs symmetrically towards the stomach. When moving, do not bend your arms at the elbows and legs at the knees. Make sure that the knees do not bend, return the legs fully extended towards the initial position as much as possible, and do not touch the floor. Perform the movement alternately, without stopping. Repeat 5 times.

1. Prone position

*Exercise 1*- Outstretched arms placed next to the body. Lift your hands slightly from the surface and, fully extended, direct them towards the center of your head (put your palms together). Return to starting position in the same way. Perform the movement alternately, without stopping. If possible, perform the exercise with weights (1 kg). Repeat 10 times.

*Exercise 2* - arms outstretched above the head, lift one arm and the opposite leg together, and alternate (Alternating Superman exercise). If possible, perform the exercise with weights (1 kg). Perform the movement alternately, without stopping. Repeat 10 times.

*Exercise 3* - arms outstretched above the head, raise the arms and legs together in an upward direction (superman exercise). If possible, perform the exercise with weights (1 kg). Perform the movement alternately, without stopping. Repeat 10 times

*Exercise 4* - leaning on your elbows, moving your head back and forth, and turning to the left and right. Hold each movement for 3 seconds and repeat 10 times.

.

1. Four-legged position

*Exercise 1* – alternately raising one arm and the opposite leg, holding the movement for 5 seconds, 10 repetitions. Hold a 1 kg weight in your hands.

*Exercise 2* - pulling the abdomen up and vice versa down (cat-cow exercise). Hold the movement for 3 seconds, 10 repetitions.

*Exercise 3* - from the four-legged position, direct the body backward, with outstretched arms and hold the position for at least 15 seconds (*Prayer Exercise*). From this position, pull the body forward, strengthening the armrests and straightening the back as much as possible. For an additional stretch, point your head back. One repeat.
