# Supplementary material for: Comparison of supervised exercise therapy with or without biopsychosocial approach for chronic nonspecific low back pain: a randomized controlled trial
Source: BMC Musculoskelet Disord. 2022 Nov 8;23:966. doi: 10.1186/s12891-022-05908-3 (PMC9641911; doi:10.1186/s12891-022-05908-3)
Supplement: Supplementary file 3 — Additional file 3. Education intervention in Graded Activity and Supervised Exercise Therapy groups. [file 12891_2022_5908_MOESM3_ESM.docx]

*Additional file 3*. Education intervention in Graded Activity and Supervised Exercise Therapy groups

At the start of interventions, the participants were provided the basic anatomy of the spine, nonspecific LBP, potential causes of nonspecific LBP, pain pathway and mechanism of chronic pain, ergonomics - proper posture in daily activities (standing, sitting, lifting, carrying, walking), and the importance of activities. Also, through education participants were informed about the many misconceptions associated with LBP as well as evidence-based treatments.

It was performed in a group during the first two sessions and lasted 10 minutes; at the participants’ request, certain contents (proper posture) were repeated in other sessions.

**Anatomy of spine**

Participants were introduced to the basic structure of our spine and the importance of the lumbar segment in coping with the load of our body during daily activities. Along with the poster presentation of the explanation is the role of the functional unit of the spine, with all its structures. Also, participants were briefly explained that the term "nonspecific" low back pain implies that it is not known exactly which part of the functional unit of the spine is responsible for their pain, but that in most cases (up to 70%) it is assumed that they are responsible deep back muscles as well as pelvis and buttocks. The cause can be either excessive load, as well as inactivity (prolonged sitting) or sudden movement/jerk of our body. However, these allegations cannot be reliably confirmed because no diagnostic test or assessment has yet been confirmed as valid. However, no serious spinal pathology is certainly the cause of their LBP ([1-4](#_ENREF_1)).

**Pain education**

In pain education, participants were briefly shown the pain pathway. The onset of pain due to some changes in the muscles (and/or functional unit of the spine) occurs due to activation of the nociceptor in that affected area. Received nociception travel and are processed in our brain, from where information is sent and a sensation of pain occurs. The role of pain is protective, as it reduces movements in the area to prevent further damage to the potential structures of our spine. However, these allegations relate more to acute pain.

In long-term pain (chronic) nociceptors can be activated without the presence of physical damage. Namely, during continuous stimuli of the nociceptor, there are changes in the processing of pain and our body responds to minor stimuli (peripheral and central sensitization). Sensory, emotional, cognitive, and behavioral elements play an important role in this process. That is, our experience and behavior regarding our pain play an important role. Avoidance of activity and prolonged rest, and fear of re-feeling a stronger intensity of pain cause the sensation of pain, as well as the consequent disability, to be constantly present. According to the latest evidence, the structures of our brain (cortico-limbic system) play a major role in the occurrence, development, duration, and intensity of chronic pain ([5-8](#_ENREF_5)).

**LBP / CNLBP misconceptions assessed and resolved**

1. Imaging is not necessary for diagnosis and treatment plan
2. Present pain is not an indicator of tissue damage, and the pain is associated with structural changes in the spine
3. Daily loading and bending are not harmful to the lumbar spine
4. Activities should not be avoided in the presence of pain; on the contrary, one should remain active as much as possible
5. LBP / CNLBP is a rarely dangerous condition (when serious pathology of the spine is the cause)
6. Weak muscles and poor posture do not have to be the cause of pain
7. Various treatments (injections, strong medications, and surgeries, various physiotherapy interventions) generally do not help
8. Various ergonomic aids (mattresses, pillows, spine holders) do not help with LBP/CNLBP
9. Exercise therapy and staying active despite pain is the most optimal solution for LBP/CNLBP

**Proper posture education**

Proper posture of the body during various daily activities adapted to the current knowledge of physiotherapists acquired during schooling and practical experience, and evidence of the etiology, risk factors, and prevention of LBP in the available literature ([9](#_ENREF_9), [10](#_ENREF_10)).

**Standing**

1. Maintaining a straight spine, avoiding side bending, hyperextension, or leaning forward

2. Keep your head upright, flush with your spine,

3. Hands placed next to the body, or on the back

4. Knees slightly bent

5. Spread legs slightly less than shoulder-width apart, heel rest;

6. While standing, hold the support on both feet (do not switch from one foot to the other), direct the main support to the heels feet;

7. Comfortable footwear, avoiding high heels, as well as completely flat shoes;

8. Avoid prolonged standing (more than 2 hours).

**Sitting**

1. While sitting, the back should be stretched and the shoulders should be pushed back. The buttocks must touch the back of the chair.
2. All three physiological planes of the spine must be present when sitting.
3. It is recommended to place the lumbar roll (or to compensate for it) between the back of the chair and the lumbar spine.
4. Legs bent at the knees at right angles, with full foot support on the ground. Keep your knees straight or slightly above your hips.
5. Distribute your body weight evenly on both hips.
6. Do not sit cross-legged.
7. Adjust the height of the chair to yourself, as well as the desk (arm bent at the elbows must touch the side back of the chair or desk).
8. When rotating movable chairs, avoid turning at the waist; the rotation is performed with the whole body.
9. Avoid prolonged sitting; recommendation to get up every 30 minutes or stretch your back (raise your arms, point backward, join your palms and turn them upwards, hold for 15 to 30 seconds; in the same position slightly point left, then right).
10. When getting up, move towards the front of the chair, keep your back straight, direct your weight to your legs and gently straighten your knees with full support on your feet (heel) to stand up. You can repeat the stretching in a standing position.

*Sitting in the car*

1. When sitting in a car, it is also recommended to use a lumbar roll, back straight, knees at right angles, in a plane, or slightly above the hips.
2. Move the seat as close to the steering wheel as possible, possible bending at the knees, and feet to the pedals.
3. Hold the steering wheel with both hands when possible.
4. When driving backward, turn the body at the waist, not just the head.

**Lifting**

1. Before lifting the object, the feet must be in a firm position on the ground.
2. When lifting objects below waist level, keep your back straight and bend at the hips and knees; Do not lift by bending forward and with outstretched knees.
3. Take a wide position next to the object to be lifted, embrace the object with both hands, tighten the abdominal muscles and lift the object with the leg muscles. Keep the object as close to the body as possible. Straighten your knees with even movements.
4. Avoid turning or twisting your body while lifting or holding a heavy object.
5. Avoid lifting objects over 11.3 kg with both hands and lifting heavier objects above shoulder level and below (up to the waist);
6. When lowering the object, place your feet firmly on the ground as if to lift, bend your hips and knees, and lower the object firmly.

**Carrying**

1. Distribute the weight of the items you are carrying on both hands.
2. Avoid major waist turns as well as body twitches.

**Pushing objects**

1. Bend your knees so that the middle of your head (ears), shoulders, and hips are in line.
2. Retract and tighten the abdominal muscles.
3. Lean towards the object, and use your legs and body strength to push the object;
4. Avoid pushing objects over 25.4 kg in weight.

**Sleeping**

1. Sleeping on your back or side is recommended. The sleeping position should allow the curves of the spine to be retained.
2. The pillow should be under the head, not the shoulders.
3. In the supine position, the height of the pillow should allow for a normal head position (head flush with the spine), and it is recommended to place the lumbar roll (or compensating object) in the lower back and the pillow below the knee.
4. In the lateral position, the knees should be slightly bent (do not draw them to the chest), and the height of the pillow under the head should correspond to the distance between the head and shoulders; it is optimal to place a pillow between the knees.
5. Getting out of bed should be done from a side position, gently pulling the knees and lowering one leg and the other to the floor, keeping the back and head flat, with firm footrests on the ground, and keeping your hands on the edge of the bed directing weight to the leg muscles and stand up.

**Walking**

1. Proper posture, as for standing, straightened spine, head in a straight raised position, shoulders down and back, activated abdominal muscles and buttocks muscles.
2. The leg that goes into the stride bends at the hip and knee, lifts, and pushes forward. During this time, the other leg maintains balance and stability using the buttocks, back, and torso muscles.
3. The lowering of the foot in the step is performed with light movements, with the primary support on the heel, and then the rest of the foot.
4. The opposite arm is directed towards the leg in the step (participants were shown how to direct the opposite arm towards the knee of the raised leg).
5. In this way, the movements of one leg and the other are performed alternately.
6. Walking is performed in light, small steps (avoid large steps), with alternating stages of oscillation and support. The width of the steps should be as small as possible, as if in a straight line; provided that this depends on the existing balance of the body.

After completing the interventions, participants in both intervention groups were advised on the importance of continuing exercise during the follow-up period. Several lying-down exercises were selected for homework: stretching exercises (knee-chest, lumbar extensor stretch, hamstrings stretch, gluteal stretch, side-to-side backstretch) and stabilization exercises (pelvic tilt, superman, lying lateral leg lifts, partial sit up). The recommended frequency of exercises was 2 times a week, and a stronger intensity, but to adjust the same to yourself. Stretching exercises (described in the proper sitting section) are recommended for daily use; after waking up, sitting for a long time, driving, or standing. They are also advised to walk, 3 times a week for about 30 minutes; with faster intensity, adhering to the learned rules of proper walking. With a note: adjust the intensity of exercise and walking to the current individual feeling.

References:

1. Morlion B. Chronic low back pain: pharmacological, interventional and surgical strategies. Nat Rev Neurol. 2013;9(8):462-73.

2. Maher C, Underwood M, Buchbinder R. Non-specific low back pain. The Lancet. 2017;389(10070):736-47.

3. Vining RD, Minkalis AL, Shannon ZK, Twist EJ. Development of an Evidence-Based Practical Diagnostic Checklist and Corresponding Clinical Exam for Low Back Pain. J Manipulative Physiol Ther. 2019;42(9):665-76.

4. Petersen T, Laslett M, Juhl C. Clinical classification in low back pain: best-evidence diagnostic rules based on systematic reviews. BMC Musculoskelet Disord. 2017;18(1):188.

5. Yang S, Chang MC. Chronic Pain: Structural and Functional Changes in Brain Structures and Associated Negative Affective States. International journal of molecular sciences. 2019;20(13).

6. Pak DJ, Yong RJ, Kaye AD, Urman RD. Chronification of Pain: Mechanisms, Current Understanding, and Clinical Implications. Curr Pain Headache Rep. 2018;22(2):9.

7. Tegner H, Frederiksen P, Esbensen BA, Juhl C. Neurophysiological Pain Education for Patients With Chronic Low Back Pain: A Systematic Review and Meta-Analysis. The Clinical journal of pain. 2018;34(8):778-86.

8. Clarke CL, Ryan CG, Martin DJ. Pain neurophysiology education for the management of individuals with chronic low back pain: systematic review and meta-analysis. Manual therapy. 2011;16(6):544-9.

9. Krishnamurthy I, Othman R, Baxter GD, Mani R. Risk factors for the development of low back pain: an overview of systematic reviews of longitudinal studies. Physical Therapy Reviews. 2018;23(3):162-77.

10. Taylor JB, Goode AP, George SZ, Cook CE. Incidence and risk factors for first-time incident low back pain: a systematic review and meta-analysis. Spine J. 2014;14(10):2299-319.
