# Supplementary material for: Comparison of supervised exercise therapy with or without biopsychosocial approach for chronic nonspecific low back pain: a randomized controlled trial
Source: BMC Musculoskelet Disord. 2022 Nov 8;23:966. doi: 10.1186/s12891-022-05908-3 (PMC9641911; doi:10.1186/s12891-022-05908-3)

*Additional file 4*. Recommendations of home-performance therapy exercises and instructions on proper posture during daily life activities in the control group


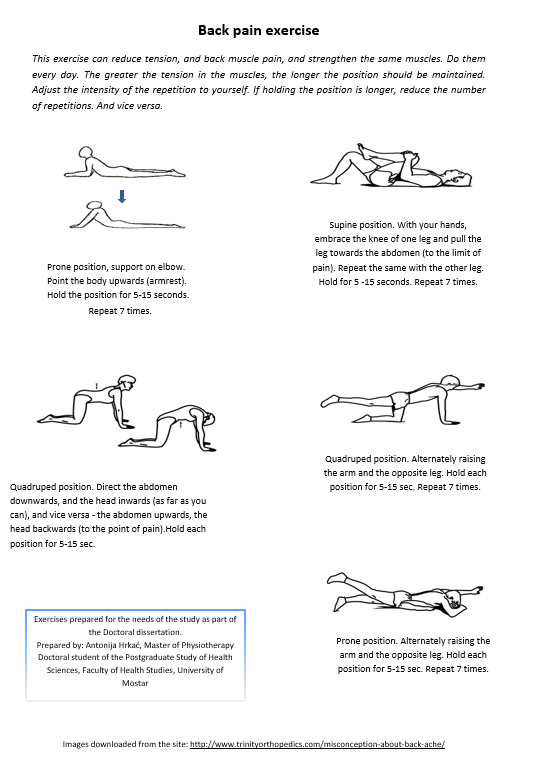


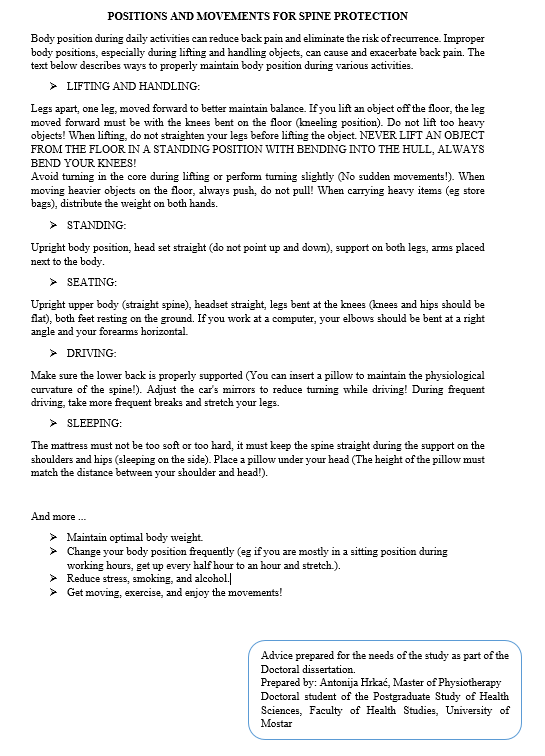

Supplement: Supplementary file 4 — Additional file 4. Recommendations of home-performance therapy exercises and instructions on proper posture during daily life activities in the control group. [file 12891_2022_5908_MOESM4_ESM.docx]
