# Supplementary material for: Comparison of supervised exercise therapy with or without biopsychosocial approach for chronic nonspecific low back pain: a randomized controlled trial
Source: BMC Musculoskelet Disord. 2022 Nov 8;23:966. doi: 10.1186/s12891-022-05908-3 (PMC9641911; doi:10.1186/s12891-022-05908-3)

*Additional file 5. a.* Sociodemographic Questionnaire


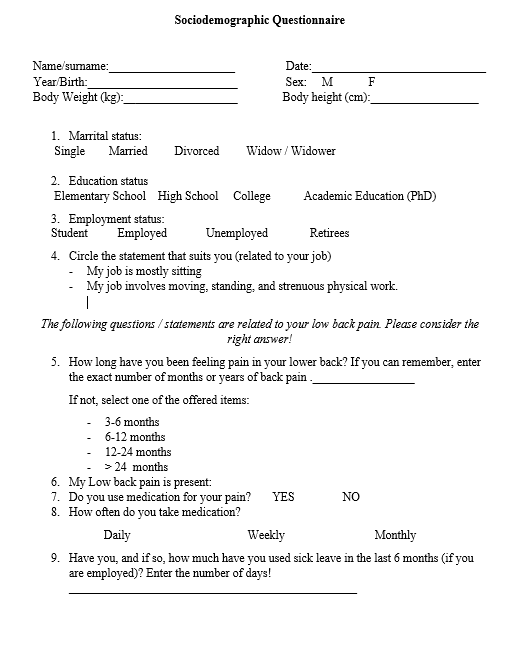


*Additional file 5. b.* Treatment Satisfaction Questionnaire


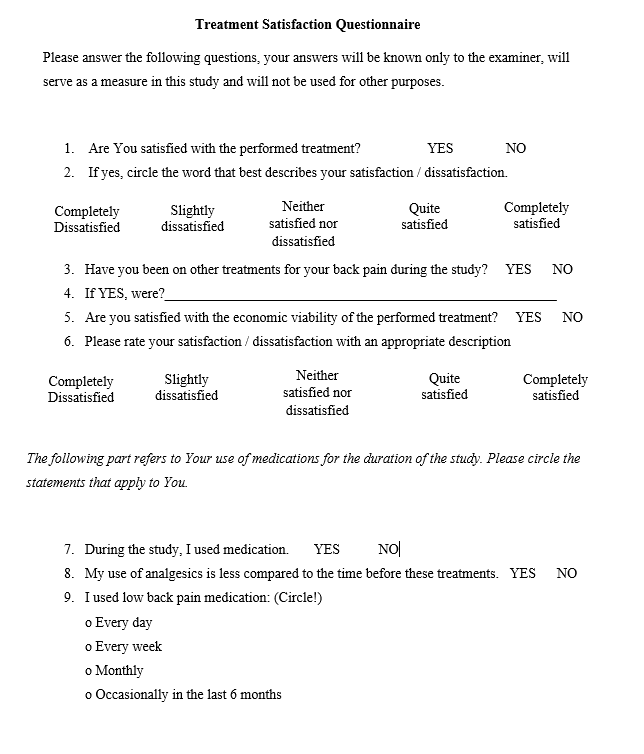

Supplement: Supplementary file 5 — Additional file 5. Sociodemographic Questionnaire and Treatment Satisfaction Questionnaire. [file 12891_2022_5908_MOESM5_ESM.docx]
