# Supplementary material for: Comparison of supervised exercise therapy with or without biopsychosocial approach for chronic nonspecific low back pain: a randomized controlled trial
Source: BMC Musculoskelet Disord. 2022 Nov 8;23:966. doi: 10.1186/s12891-022-05908-3 (PMC9641911; doi:10.1186/s12891-022-05908-3)
Supplement: Supplementary file 6 — Additional file 6. Outcome measurement instruments. [file 12891_2022_5908_MOESM6_ESM.docx]

*Additional file 6*. Outcome measurement instruments

*The socio-demographic questionnaire,* designed for this study, was used to collect personal, socio-demographic information about participants. An example of a questionnaire is shown in Supplementary file 4.

*Visual Analog Scale* (VAS) is a one-dimensional instrument for the subjective assessment of pain intensity in a patient, used in a variety of pain conditions. It consists of a line 100 mm long. The beginning of the line (corresponding to the 0) indicates the absence of pain, and the end of the line (corresponding to the number 100) indicates the worst possible pain. The scale is scored by measuring the distance from the start of the line, which indicates “no pain” to the point indicated by the patient. The patient independently indicates which distance from the start of the line best describes the severity of their pain. We can interpret VAS according to the usual distribution of pain into no pain, mild pain, moderate pain, and severe pain, most commonly described by patients. Thus, a score on the VAS of 0 to 4 mm indicates no pain, 5 to 44 mm indicates mild pain, 45 to 74 mm moderate pain, and 75 to 100 mm severe pain. The advantage of this instrument is its intelligibility and simplicity, it is easy to explain to the patient and thus does not take much time to complete (˂ 1 minute). VAS is a well-studied method for measuring pain intensity in acute and chronic back pain, and its validity has been confirmed by chronic musculoskeletal pain ([1](#_ENREF_1), [2](#_ENREF_2)).

*The Roland-Morris Disability Questionnaire* (RMDQ) is a valid and reliable instrument for the assessment of functional disability in LBP. The original version was published in 1983 by Roland and Morris and was updated in 2000 ([3](#_ENREF_3), [4](#_ENREF_4)). The updated Croatian version was used in this study, available at <http://www.rmdq.org/download.htm>. It consists of 24 questions that assess the daily functional impact of low back pain (mobility, activities and daily life) and is scored by a total of 0 to 24 points. A higher sum indicates a higher disability.

The RMDQ is a well-established measure and is widely used in clinical trials. The Initiative on Methods, Measurement, and Pain Assessment in Clinical Trials (IMMPACT) guidelines states the RMDQ is an example of a disease-specific measure that has been developed to evaluate physical function in LBP, as well as CLBP. RMDQ has a well-documented evaluation of psychometric properties. In the test-retest reliability of 24-item RMDQ intraclass correlation (ICC) ranges from 0.42 - 0.91. The construct validity study showed a good correlation with pain scores, and physical subscales of SF-36 and the weakest correlations were reported with objective tests of physical function such as finger–to–floor and straight leg raise. The RMDQ has content validity and is considered a reliable and valid tool to assess the impact of CLBP on physical functioning ([4-7](#_ENREF_4)).

*Range of Movements* (ROM) was measured with a goniometer (extension) and Finger-to-Floor test (anteflexion).

Goniometer - In this study, measurements were performed using a simple goniometer; The participant was in an upright starting position with the knees fully extended and the arms behind the neck. The goniometer axis is placed on the crista iliac. The participant was in an upright starting position with the knees fully extended and the arms behind the neck. The goniometer axis is placed on the crista iliac crest, aligned with the midaxillary line. The participant was instructed to slowly and gradually bend directly backward, as far as possible, without bending the knees. The participant repeats the extension movement twice, and the maximum range of extension is taken as a measure. All ROM measurements were taken in the afternoon. Measurements are recorded in degrees (°)The normative value of the extension movement in the lumbar spine is ≥ 30° ([8](#_ENREF_8), [9](#_ENREF_9)).

Finger-to-Floor Distance (FTF, Thomayer's Test) - Functional measurement where finger-to-floor distance is recorded using a centimeter strip in spine flexion. The finger-to-floor distance is a reliable and valid measurement method that can be used in clinical practice and research into the effectiveness of therapeutic interventions. The participants are in a standing position, with the knees fully extended and the extended hand. The participant received verbal instructions: “Now bend forward with hands extended, while not bending your knees and try to touch the floor with your fingers if you can". The participant repeated the bending movement three times (without taking a measurement), and then performed the maximum movement and kept it. The outcome assessor measured the distance of the tip of the middle finger right hand from the floor using a centimeter strip. All measurements were taken in the afternoon, at the same time. Results are expressed in centimeters (cm) ([10](#_ENREF_10)).

*Prone Double Straight Leg Raise Test* (PDSRT) – was used to evaluate the strength of the spinal extensor. It was described by McIntosh et al in 1998 in a study that obtained normative values for adults between the ages of 19 and 80. Normative values are divided into age groups of 10 years and by sex. This test has higher sensitivity, specificity, and predictive values for back pain compared to the Biering-Sorensen test, which is the more commonly used test in clinical trials for back pain ([11](#_ENREF_11)). Also, the advantage of this test is its much simpler and less expensive application (it does not require accessories) and, most importantly, it is acceptable to the population suffering from chronic back pain. The measurement procedure is performed in such a way that the subject is in a pronated (abdominal) position with his legs extended. The shoulders are vertically positioned relative to the body, with the arms below the forehead. Participants are asked to lift their outstretched legs upwards and maintain this position as far as possible. Time is measured in seconds ([12](#_ENREF_12)).

*The Fear-Avoidance Beliefs Questionnaire* (FABQ) is a questionnaire that measures a patient's fear of pain and the consequences of avoiding physical activity due to that fear, leading to an increase in negative physical and psychological effects due to back pain. The association between fear/avoidance of movement and chronic back pain is strong, and this questionnaire, as a measure of outcome in the case of chronic back pain, serves to tailor interventions, meet patient needs, and prevent further long-term disabilities. It consists of 16 items, and each is rated with scores from 0 to 6. A higher score indicates a greater fear/avoidance presence. The FABQ is divided into two units (work and physical activity), one measuring the association of work with the current feeling of pain in the back pain and the other the correlation of physical activity with the current feeling of pain in the back pain ([13](#_ENREF_13)). FABQ is a valid and reliable chronic back pain questionnaire; the total FABQ test-retest reliability is 0.97, FABQ Physical Activity subscale test-retest reliability ranged from 0.72-0.90, and FABQ Work subscale test-retest reliability ranged from 0.80-0.91. The validity assessment found a correlation between FABQ and the Roland and Morris Disability Questionnaire. The correlation coefficients for the FABQ, the FABQ Work subscale and the FABQ Physical Activity subscale are 0.52, 0.63, and 0.51 ([14](#_ENREF_14)). In this study, we used the Croatian version ([15](#_ENREF_15)).

*The Hospital Anxiety and Depression Scale* (HAD) is a self-rating scale used widely in health care settings and clinical research to assess symptoms of depression and anxiety. It consists of 14 questions, 7 questions for depression assessment, and 7 for anxiety assessment, and the question and answer period is the past week. The answers are scored in four levels from 0 to 3 (0 = not at all, 3 = all the time). The total score can range from 0 to 21 for depression or anxiety. Individuals with a score of 0-7 are not depressed/anxious, 8-10 indicate a borderline condition and 11-21 represent depression/anxiety. Because of the division into two subscales of depression and anxiety, it is a valid and reliable instrument in chronic pain studies ([16](#_ENREF_16), [17](#_ENREF_17)). In this study, we used the available Croatian version.

*The Health Survey* (SF 36) is the most commonly used questionnaire to assess the quality of life associated with health status; It is valid for use in the Croatian population ([18](#_ENREF_18)). In this study, because of the ease of access to the participants, we used a shorter version of the questionnaire (SF 12), consisting of 12 questions: two on physical functioning, 2 on the role of physical functioning, one on the physical pain, one on general health, one of the vitality, 1 on social functioning, 2 on the emotional role, and 2 on mental health. The SF 12 has good internal consistency reliability, construct validity, and responsiveness in patients with back pain ([19](#_ENREF_19)). With this questionnaire it is possible to quantitatively compare each dimension of health, as the points for each question are converted to standard values and set on a scale from 0 to 100 (a higher score is better health), thus obtaining two main dimensions of health, physical and mental health (Physical Common Score-PCS and Mental Common Score-MCS) ([16](#_ENREF_16), [20](#_ENREF_20)).

*Treatment Satisfaction Questionnaire* is a self-structured self-assessment questionnaire for measuring participants' satisfaction with the implemented interventions, as well as their economic cost-effectiveness. The assessment of satisfaction with interventions as well as cost-effectiveness was measured using the 5-item Likert scale, from description completely dissatisfied to completely satisfied. Also, this questionnaire contained questions about the frequency of medication use during the study, as well as possible participation in other treatments due to low back pain during the study. The questionnaire in this study was prepared according to the recommendations of the scientific literature ([5](#_ENREF_5), [21](#_ENREF_21)).

**References:**

1. Boonstra AM, Schiphorst Preuper HR, Reneman MF, Posthumus JB, Stewart RE. Reliability and validity of the visual analogue scale for disability in patients with chronic musculoskeletal pain. International journal of rehabilitation research Internationale Zeitschrift fur Rehabilitationsforschung Revue internationale de recherches de readaptation. 2008;31(2):165-9. doi:10.1097/MRR.0b013e3282fc0f93

2. McCormack HM, Horne DJ, Sheather S. Clinical applications of visual analogue scales: a critical review. Psychological medicine. 1988;18(4):1007-19. doi:10.1017/s0033291700009934

3. Roland M, Morris R. A study of the natural history of back pain. Part I: development of a reliable and sensitive measure of disability in low-back pain. Spine (Phila Pa 1976). 1983;8(2):141-4. doi:10.1097/00007632-198303000-00004

4. Roland M, Fairbank J. The Roland-Morris Disability Questionnaire and the Oswestry Disability Questionnaire. Spine (Phila Pa 1976). 2000;25(24):3115-24. doi:10.1097/00007632-200012150-00006

5. Dworkin RH, Turk DC, Farrar JT, Haythornthwaite JA, Jensen MP, Katz NP, et al. Core outcome measures for chronic pain clinical trials: IMMPACT recommendations. Pain. 2005;113(1-2):9-19. doi:10.1016/j.pain.2004.09.012

6. Burbridge C, Randall JA, Abraham L, Bush EN. Measuring the impact of chronic low back pain on everyday functioning: content validity of the Roland Morris disability questionnaire. Journal of patient-reported outcomes. 2020;4(1):70. doi:10.1186/s41687-020-00234-5

7. Macedo LG, Maher CG, Latimer J, Hancock MJ, Machado LA, McAuley JH. Responsiveness of the 24-, 18- and 11-item versions of the Roland Morris Disability Questionnaire. Eur Spine J. 2011;20(3):458-63. doi:10.1007/s00586-010-1608-2

8. Fitzgerald GK, Wynveen KJ, Rheault W, Rothschild B. Objective assessment with establishment of normal values for lumbar spinal range of motion. Phys Ther. 1983;63(11):1776-81. doi:10.1093/ptj/63.11.1776

9. Chertman C, Campoy Dos Santos HM, Pires L, Wajchenberg M, Martins DE, Puertas EB. A Comparative Study of Lumbar Range of Movement in Healthy Athletes and Non-Athletes. Rev Bras Ortop. 2010;45(4):389-94. doi:10.1016/S2255-4971(15)30385-2

10. Perret C, Poiraudeau S, Fermanian J, Colau MM, Benhamou MA, Revel M. Validity, reliability, and responsiveness of the fingertip-to-floor test. Arch Phys Med Rehabil. 2001;82(11):1566-70. doi:10.1053/apmr.2001.26064

11. McIntosh G WL, Affieck M, Hall H. Trunk and lower extremity muscle endurance: normative data for adults 1998. Available from: https://[www.researchgate.net/publication/233783627_Trunk_and_lower_extremity_muscle_endurance_Normative_data](http://www.researchgate.net/publication/233783627_Trunk_and_lower_extremity_muscle_endurance_Normative_data).

12. Arab AM, Salavati M, Ebrahimi I, Ebrahim Mousavi M. Sensitivity, specificity and predictive value of the clinical trunk muscle endurance tests in low back pain. Clin Rehabil. 2007;21(7):640-7. doi:10.1177/0269215507076353

13. Waddell G, Newton M, Henderson I, Somerville D, Main CJ. A Fear-Avoidance Beliefs Questionnaire (FABQ) and the role of fear-avoidance beliefs in chronic low back pain and disability. Pain. 1993;52(2):157-68. doi:10.1016/0304-3959(93)90127-b

14. Williamson E. Fear Avoidance Beliefs Questionnaire (FABQ). The Australian journal of physiotherapy. 2006;52(2):149. doi:10.1016/s0004-9514(06)70052-6

15. Đorđić J. Uloga kognitivnog stila obilježenog strepnjom i anksiozne osjetljivosti u razvoju boli i različitih aspekata bolnog iskustva tijekom fizikalne terapije. Specijalistički rad. Zagreb: Filozofski fakultet u Zagrebu, Odsjek za psihologiju.; 2018. Available from: <http://darhiv.ffzg.unizg.hr/id/eprint/10165/1/Dordic_Jelena.pdf>.

16. LoMartire R, Äng BO, Gerdle B, Vixner L. Psychometric properties of Short Form-36 Health Survey, EuroQol 5-dimensions, and Hospital Anxiety and Depression Scale in patients with chronic pain. PAIN. 2020;161(1).

17. Castro MM, Quarantini L, Batista-Neves S, Kraychete DC, Daltro C, Miranda-Scippa A. [Validity of the hospital anxiety and depression scale in patients with chronic pain.]. Rev Bras Anestesiol. 2006;56(5):470-7. doi:10.1590/s0034-70942006000500005

18. Maslic Sersic D, Vuletic G. Psychometric evaluation and establishing norms of Croatian SF-36 health survey: framework for subjective health research. Croat Med J. 2006;47(1):95-102.

19. Luo X, George ML, Kakouras I, Edwards CL, Pietrobon R, Richardson W, et al. Reliability, validity, and responsiveness of the short form 12-item survey (SF-12) in patients with back pain. Spine (Phila Pa 1976). 2003;28(15):1739-45. doi:10.1097/01.BRS.0000083169.58671.96

20. Busija L, Pausenberger E, Haines TP, Haymes S, Buchbinder R, Osborne RH. Adult measures of general health and health-related quality of life: Medical Outcomes Study Short Form 36-Item (SF-36) and Short Form 12-Item (SF-12) Health Surveys, Nottingham Health Profile (NHP), Sickness Impact Profile (SIP), Medical Outcomes Study Short Form 6D (SF-6D), Health Utilities Index Mark 3 (HUI3), Quality of Well-Being Scale (QWB), and Assessment of Quality of Life (AQoL). Arthritis Care Res (Hoboken). 2011;63 Suppl 11:S383-412. doi:10.1002/acr.20541

21. Bombardier C. Outcome assessments in the evaluation of treatment of spinal disorders: summary and general recommendations. Spine (Phila Pa 1976). 2000;25(24):3100-3. doi:10.1097/00007632-200012150-00003
